# Probing stress-regulated ordering of the plant cortical microtubule array via a computational approach

**DOI:** 10.1186/s12870-023-04252-5

**Published:** 2023-06-09

**Authors:** Jing Li, Daniel B. Szymanski, Taeyoon Kim

**Affiliations:** 1 Weldon School of Biomedical Engineering, Purdue University, 206 S Martin Jischke Dr, West Lafayette, IN 47907 USA; 2 Botany and Plant Pathology, Biological Sciences, Purdue University, 915 West State Street, West Lafayette, IN 47907 USA

**Keywords:** Plant cortex, Microtubule, Ordering, Alignment, Stress

## Abstract

**Background:**

Morphological properties of tissues and organs rely on cell growth. The growth of plant cells is determined by properties of a tough outer cell wall that deforms anisotropically in response to high turgor pressure. Cortical microtubules bias the mechanical anisotropy of a cell wall by affecting the trajectories of cellulose synthases in the wall that polymerize cellulose microfibrils. The microtubule cytoskeleton is often oriented in one direction at cellular length-scales to regulate growth direction, but the means by which cellular-scale microtubule patterns emerge has not been well understood. Correlations between the microtubule orientation and tensile forces in the cell wall have often been observed. However, the plausibility of stress as a determining factor for microtubule patterning has not been directly evaluated to date.

**Results:**

Here, we simulated how different attributes of tensile forces in the cell wall can orient and pattern the microtubule array in the cortex. We implemented a discrete model with transient microtubule behaviors influenced by local mechanical stress in order to probe the mechanisms of stress-dependent patterning. Specifically, we varied the sensitivity of four types of dynamic behaviors observed on the plus end of microtubules – growth, shrinkage, catastrophe, and rescue – to local stress. Then, we evaluated the extent and rate of microtubule alignments in a two-dimensional computational domain that reflects the structural organization of the cortical array in plant cells.

**Conclusion:**

Our modeling approaches reproduced microtubule patterns observed in simple cell types and demonstrated that a spatial variation in the magnitude and anisotropy of stress can mediate mechanical feedback between the wall and of the cortical microtubule array.

**Supplementary Information:**

The online version contains supplementary material available at 10.1186/s12870-023-04252-5.

## Background

Morphogenesis of plant cells and tissues requires coordinated shape changes among adherent cells that do not migrate relative to one another [1, 2]. Although the turgor pressures that drive expansion are isotropic, plant cells can grow in a polarized fashion since the cell wall often displays highly anisotropic stiffness [3–5]. Cellulose-dependent wall anisotropy underlies morphological diversity in the plant kingdom [6], and the orientation of the cellulose microfibrils is determined in large part by the cortical microtubule cytoskeleton [7–9]. Cortical microtubules are tightly coupled to the plasma membrane [10] and bias the directions of active cellulose synthase (CESA) complexes [3, 7]. The cell wall becomes stiffer in a direction in which the cellulose fibers are synthesized and oriented because of their high axial stiffness and strong lateral interactions between microfibrils [5], so elongation is restricted in that dimension. A major challenge in the field of plant development is to understand how cortical microtubule arrays are structurally organized relative to the geometry of cells and tissues during morphogenesis.

Microtubules are cytoskeletal polymers composed of α and β tubulins and have polarity defined by plus and minus ends with unique biochemical and kinetic properties [11, 12]. Microtubules are highly dynamic polymers; individual microtubules and an entire microtubule network turn over on the time scale of minutes [13–15]. The underlying dynamic instability of microtubules mediates these behaviors [13, 14, 16, 17]. The nucleation of microtubules in the plant cortex can occur de novo or via branching from pre-existing microtubules [18–20]. Although both ends of microtubules exhibit a frequent switch between growth, shrinkage, and pause states (i.e., three-state dynamics) [11], the plus end is far more dynamically unstable than the minus end. When microtubules in the plant cortex grow from the plus end, they often encounter other microtubules since polymer dynamics of microtubules tightly coupled to the plasma membrane takes place on two-dimensional (2D) surfaces [10]. Collisions between microtubules can lead to different consequences depending on the angle of collision [16]. When the plus end of a microtubules makes contact to other microtubule with an angle below ~ 40˚, its polymerization is reoriented in a direction parallel to the other microtubule, which is referred to as zippering. By contrast, a collision with a contact angle greater than ~ 40˚ leads to either crossover or a transition from growth to shrinkage, which is called catastrophe. The collision-induced catastrophe and zippering are known to mediate ordered cortical microtubule arrays emerging at cellular length-scales. It was shown that a balance between steep-angle catastrophe and shallow-angle zippering is necessary and sufficient for promoting self-organization and parallel configuration of microtubules [16, 21]. Regardless of collisions, microtubules undergoing rapid shrinkage can transition to a growth state, which is called rescue.

The constricted regions of lobed pavement cells are emphasized as locations for microtubule alignment [22, 23], and quantitative analyses have detected subtle enrichments of transfacial microtubules at subsets of cell indentations [24–26]. Stresses in the anticlinal wall that are orthogonal to the epidermal surface can strongly orient microtubules in this cell face [15, 27]. Polarized trichoblasts like leaf hairs and cotton fibers present a simpler case since they lack neighboring cells and biomechanically distinct cell faces. During their anisotropic growth phases, transverse microtubules are present along the cell flank distal to the apical microtubule depletion zone (MDZ) [28–31]. Because cortical microtubules define the pattern of extracellular cellulose microfibrils in aerial trichoblasts [32], finite element (FE) models predict strict thresholds for transverse microtubules in order to enable highly anisotropic cell elongation in the absence of radial swelling [32, 33]. The apical dome of these cells is predicted to be isotropic since the MDZ has no detectable microtubule organization, and the resulting cellulose fibers would be randomly arranged. The size of the apical zone defines the radius of curvature of the cell apex as it expands [32]. At present, the mechanisms that define the cellular-scale orientations of microtubules in plant cells are poorly understood in general.

Computational modeling of microtubule array alignment is an effective strategy to analyze what parameters promote alignment of microtubule and orient the microtubules with respect to geometric features of the cell. Dixit and Cyr first introduced a Monte Carlo simulation for cortical microtubule arrays and highlighted the importance of zippering and collision-induced catastrophe for forming an aligned microtubule array [16]. Mechanisms to orient the array relative to cellular features were analyzed in a subsequent 2D model; orientations in a specific direction could occur if orthogonal cell surfaces contain a catastrophe-inducing boundary. Such a boundary effect was also observed in a three-dimensional (3D) model where the end walls (top and bottom boundaries) in a cylindrical domain act as either the catastrophe-inducing or reflective boundary [34]. Both types of the boundary condition induced transverse alignment even if collision-induced catastrophe between microtubules was disabled. A recent 3D model by Chakrabortty et al. showed that a catastrophe-inducing boundary and cell-face-specific microtubule-destabilizing surfaces can potently generate oriented microtubule arrays [23].

However, the prevalence of catastrophe-inducing boundaries and the mechanistic underpinnings of cell-face-specific control of microtubule behaviors are not well established. For example, in some epidermal cell types, microtubule destabilization was observed in highly curved regions whose radius of curvature was smaller than 2.5 μm [35]. However, tapering trichoblasts have stable microtubules in regions whose radius of curvatures is significantly smaller than 2.5 μm [32]. In addition, in epidermal cells, microtubules are often aligned along the anticlinal walls perpendicular to the cell edge [15, 27, 36]. The orientation of this microtubule alignment is orthogonal to the orientation that would be expected to appear due to a strong catastrophe-inducing boundary existing at the interface of the outer periclinal and anticlinal walls. In addition, lack of a correlation between microtubule orientation and cell geometry can be seen in epidermal cells that exhibit multiple types of microtubule configurations on the outer cell cortex over time despite their similar cell shapes [37], and different faces of the cell can simultaneously display distinct orientations [15, 38].

A correlation between mechanical stress, self-organization of cortical microtubule array, and morphogenesis has been analyzed for decades [39, 40]. Numerous studies have shown that microtubules can orient in parallel to predicted patterns of cell wall stress, and remodeling of microtubules often occurs gradually and continuously in response to perturbations that are likely to alter stress distributions [15, 22, 41–44]. Based on FE model stress predictions, the density of cortical microtubules and/or their alignment are correlated with the magnitude and direction of wall stress [15, 22, 26, 32, 33]. The mechanism by which microtubules align with stress is a black box [45, 46]. The activity could be mediated via receptor-mediated force coupling between the wall and microtubules; however, the range of stress on the cell wall that we estimated from the previous FE model is 3–15 MPa [32]. If microtubules feel this large stress directly through a contact area of ~ 300 nm^2^ on the end or side of a microtubule (for the end assume an individual microtubule acts as a hollow cylinder, then the cross section consists of 13 circular protofilaments), the magnitude of forces from the normal stress is far beyond level that microtubules can sustain without rupturing [47]. Thus, it is expected that microtubules are partially or indirectly coupled to the load-bearing molecules in the wall. The ability of microtubules to sense tensile forces has been shown in the functional attributes of the mitotic spindle [48] and the enhanced polymerization of microtubules in the in vitro reconstitution experiments [49–51]. It was also shown that suppressed depolymerization, catastrophe and enhanced rescue frequencies scale with tensile force [50].

Based on known interactions between tensile forces and microtubule dynamics, we created a discrete model with transient behaviors of microtubules influenced by local mechanical stress. By exploring a wide parametric space and imposing physiologically relevant stress patterns, we defined a plausible constitutive relationship between local stress and microtubule dynamics. We adopted stress field data imported from validated FE models to a 2D microtubule simulation domain. We found that spatial patterns of stress directionality, magnitude, and anisotropy mimicking stress distribution on realistic cell walls lead to microtubule arrays commonly observed near the cell apex. Results in our study provide a more systematic understanding of how microtubules are self-organized by responding to physiologically relevant mechanical stimuli.

## Results

Our goal is to determine whether realistic stress patterns in the wall could act as a cellular-scale cue that can mediate microtubule array organization, using a computational model. In the model, microtubules are simulated as rigid polymers with distinct dynamic behaviors of the plus and minus ends. The dynamic behaviors of each end have been measured directly using time-lapsed live cell imaging [11]. As plant microtubules are physically associated with the plasma membrane [10], a 2D model can accurately represent stochastic behaviors of microtubules and interactions between microtubules in the presence of the stress pattern [10]. Stress patterns in cell wall are quite distinct, depending on the geometry of the cell or tissue organization and cell wall material properties [15, 32, 52, 53]. We implemented stress patterns with various magnitudes and anisotropy. To generate a realistic distribution of stresses, we imported the maximum principal stress values from the validated FE model of a polarized plant cell [32]. The means by which stress is coupled to microtubules is not known. We hypothesize the existence of a population of stress-sensing components in mechanotransduction pathway that directionally couple stress to altered microtubule dynamics. Our model employs linear functions to relate stress to changes in dynamic behaviors of microtubule. Then, we analyzed how these different parameters affect the degree of microtubule alignment and the extent to which stress variability can align microtubule network.

### Anisotropic stress patterns can align microtubules by affecting microtubule dynamics

**Fig. 1:**
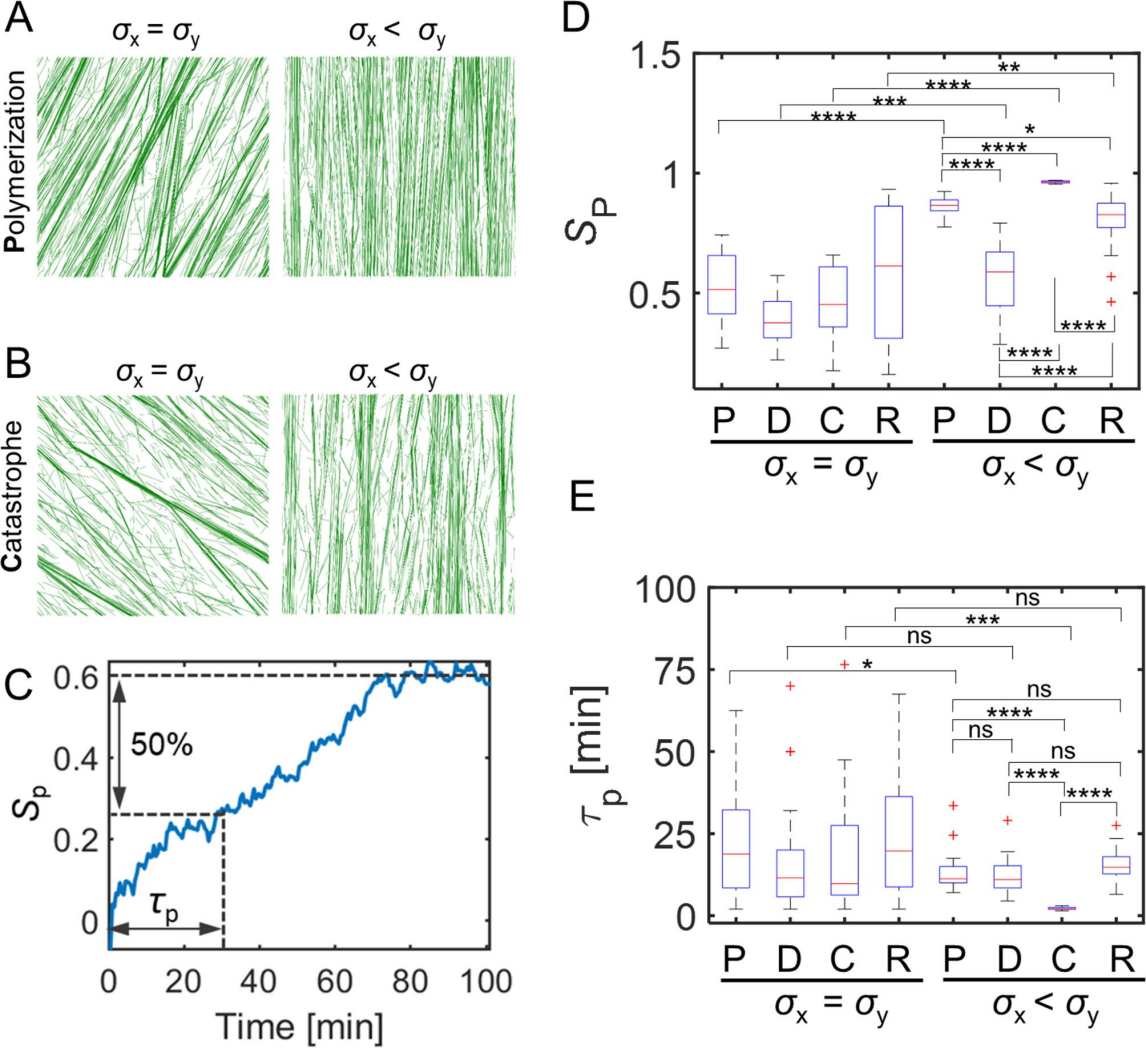
Anisotropic stress affects self-organization of microtubules and correlates with global orientation. (**A** and **B**) Steady state MT network morphology. All snapshots are taken at 100 min. MTs are subject to network stress predominant in y direction (right), isotropic (left). In **A** and **B**, polymerization rate is enhanced, and catastrophe frequency is reduced in alignment with principal stress, respectively. (**C**) A representative case with the time evolution of the network order parameter *S*_p_. The first time constant is calculated as the time required to reach half of the *S*_p_ value at steady state. In cases with isotropic stress, *S*_p_ was calculated with respect to the average orientation of all microtubules, whereas it was calculated with respect to the principal direction of stress in cases with anisotropic stress. (**D**) Summary of the network order parameter *S*_p_ at 100 min for all different conditions with isotropic vs. anisotropic stress in which principal stress influences individual stochastic parameter independently. (**E**) Boxplot of the time constant (*τ*_p_) acquired from cases with isotropic or anisotropic stress under all conditions. *τ*_p_ is time required for *S*_p_ to reach half of its steady-state value. Data for each condition are averaged over 20 simulations in each case (n = 20). P: polymerization, D: depolymerization, C: catastrophe, R: rescue; ns p > 0.05, * p < 0.05, ** p < 0.01, *** p < 0.001, **** p < 0.0001

We first tested whether or not stress anisotropy could bias the cellular-scale organization of the microtubule array. Based on experimental observations, we assume that stress enhances the polymerization rate and the rescue frequency and reduces the depolymerization rate and the catastrophe frequency (Figs. S1A, B). If the stress is isotropic, microtubule bundles align in random directions because microtubules are subjected to the same level of stress regardless of their orientations (Fig. 1A, B left and S2A, B left). Such random alignments of microtubules with isotropic stress were observed, regardless of the size of domain (Figs. S3A, B). To evaluate the extent of microtubule alignment, the order parameter (*S*_p_) was calculated with respect to the average orientation of all microtubules (Fig. 1C). If all microtubules are oriented in one direction, *S*_p_ becomes 1. *S*_p_ measured at a steady state was ~ 0.4–0.5 and was not dependent highly on which dynamic parameter was affected by stress (Fig. 1D, S3C, D). As another measure to analyze the sensitivity of different microtubule parameters to stress, the half-time to reach steady state in *S*_p_ (*τ*_p_) was estimated (Fig. 1C). *τ*_p_ did not vary significantly by different conditions when the four parameters were varied in response to an isotropic stress (Fig. 1E). The lifetime and length of microtubules were not enhanced by isotropic stress, compared to the case without stress (Table 1). When anisotropic stress with a 10:1 ratio (*σ*_x_ < *σ*_y_) was imposed, microtubules were aligned in the principal direction of stress to different extents depending on which parameter was modulated by stress (Fig. 1A, B right and S2A, B center). In these cases with anisotropic stress, the extent of alignment was evaluated by calculating *S*_p_ with respect to the principal direction of stress. Note that the anisotropy ratio was consistent with the change in tensile loads from experiments [50]. In all cases, *S*_p_ increased fast at early time points and slowed down later (Fig. S2C). Anisotropic stress increased *S*_p_ more in all cases with a stress-dependent variation in the plus end behaviors, compared to that with the isotropic stress. However, the largest increase in *S*_p_ was observed when stress enhanced the polymerization rate or reduced the catastrophe frequency (Fig. 1D). Stress-dependent modulation of the depolymerization rate was the least potent in terms of effects on *S*_p_. *τ*_p_ did not differ significantly when the depolymerization rate and the rescue frequency were modulated by stress (Fig. 1E and S2E, F). However, *τ*_p_ was significantly reduced in both cases with the polymerization rate and the catastrophe frequency varied by stress. The average length and lifetime of microtubules were also enhanced more in those cases (Fig. S2D and Table 1). Larger changes in *S*_p_, *τ*_p_, and the length and lifetime of microtubules imply that the polymerization rate and the catastrophe frequency were more efficient stress-sensitive regulators of microtubule alignment. Note that the average length of microtubules in our simulations was in good agreement with the reported average length of single cortical microtubules in plant cells, 2–4 μm [54].

**Table 1:** Average microtubule (MT) lifetime and length under various stress conditions

*τ*_life_ [min] *L*_MT_[µm]		P Polymerization	D Depolymerization	C Catastrophe	R Rescue
Stress free control	*τ*_life_	2.2 min	2.2 min	2.2 min	2.2 min
	*L*_MT_	2.0 μm	2.0 μm	2.0 μm	2.0 μm
Isotropic stress *σ*_x_ = *σ*_y_	*τ*_life_	2.3 min	2.3 min	2.3 min	2.3 min
	*L*_MT_	2.6 μm	2.0 μm	2.5 μm	2.2 μm
Anisotropic stress *σ*_x_ < *σ*_y_ or *σ*_x_ > *σ*_y_	*τ*_life_	2.9 min	2.4 min	3.0 min	2.4 min
	*L*_MT_	2.8 μm	2.2 μm	2.9 μm	2.3 μm

We next attempted to determine the threshold of stress anisotropy beyond which microtubule ordering is increased significantly. By imposing different level of stress anisotropy (*σ*_y_ / *σ*_x_), we found that the ratio of 1.5:1 was required to induce microtubule alignment in the direction of principal stress (Fig. S4). In response to stress anisotropy with the minimal ratio, both *S*_p_ (Figs. S4A, C, E, G) and τ_p_ (Figs. S4B, D, F, H) exhibited a substantial increase or decrease, respectively (p < 0.001, n = 10). Previously, the validated FE model of a growing trichome branch showed that the estimated stress anisotropy is characterized by the ratio of ~ 2:1, which reflects the anisotropy of a cylindrical shell and is shown to be associated with transverse microtubule bands [32]. This specific ratio is greater than the critical threshold (1.5:1) we found in simulations. This indicates that the stress anisotropy in the trichoblast FE model is sufficient for causing microtubule alignment.

Our results demonstrated that microtubule alignment can emerge at the scale of the stress field if the directionality of a stress field affects stochastic dynamic behaviors occurring at the plus end of microtubules, following the linear constitutive relationship. However, we observed that there is a difference in the extent of microtubule alignment in the patterns, depending on which dynamic parameter was affected. We do not want to neglect the effect of collision-induced catastrophe as it contributes to the turnover of discordant microtubules and can promote alignment of the cortical array. Therefore, we explored a 2D parametric space including stress anisotropy and collision-induced catastrophe. We found that stress anisotropy and collision-induced catastrophe can coregulate microtubule ordering (Supplementary Text and Fig. S5).

### Stress reorientation can reorganize aligned microtubule patterns

Stresses that influence microtubule organization are affected by tissue-level tension and the growth states of underlying cells [42, 55]. Therefore, stress patterns are not static features but can change at different time scales. As such, dynamic stress patterns can lead to the reorientation of cortical microtubule array [42, 43]. To determine whether an established ordered microtubule array can be reoriented by a change in stress anisotropy, we imposed a dynamic stress pattern that is dominant in y direction at the beginning for 100 min and then undergoes a directional shift at the mid of the simulation. We assumed that the catastrophe frequency is affected by the time-varying stress because it was found to be the most efficient stress-sensitive parameter for microtubule alignment. A transition in the stress pattern occurred either instantaneously or gradually to reflect the slow shape change of plant cells that occurs on timescales of tens of minutes. The ratio of stress was 2:1 both at the beginning and at end of the transition.

**Fig. 2:**
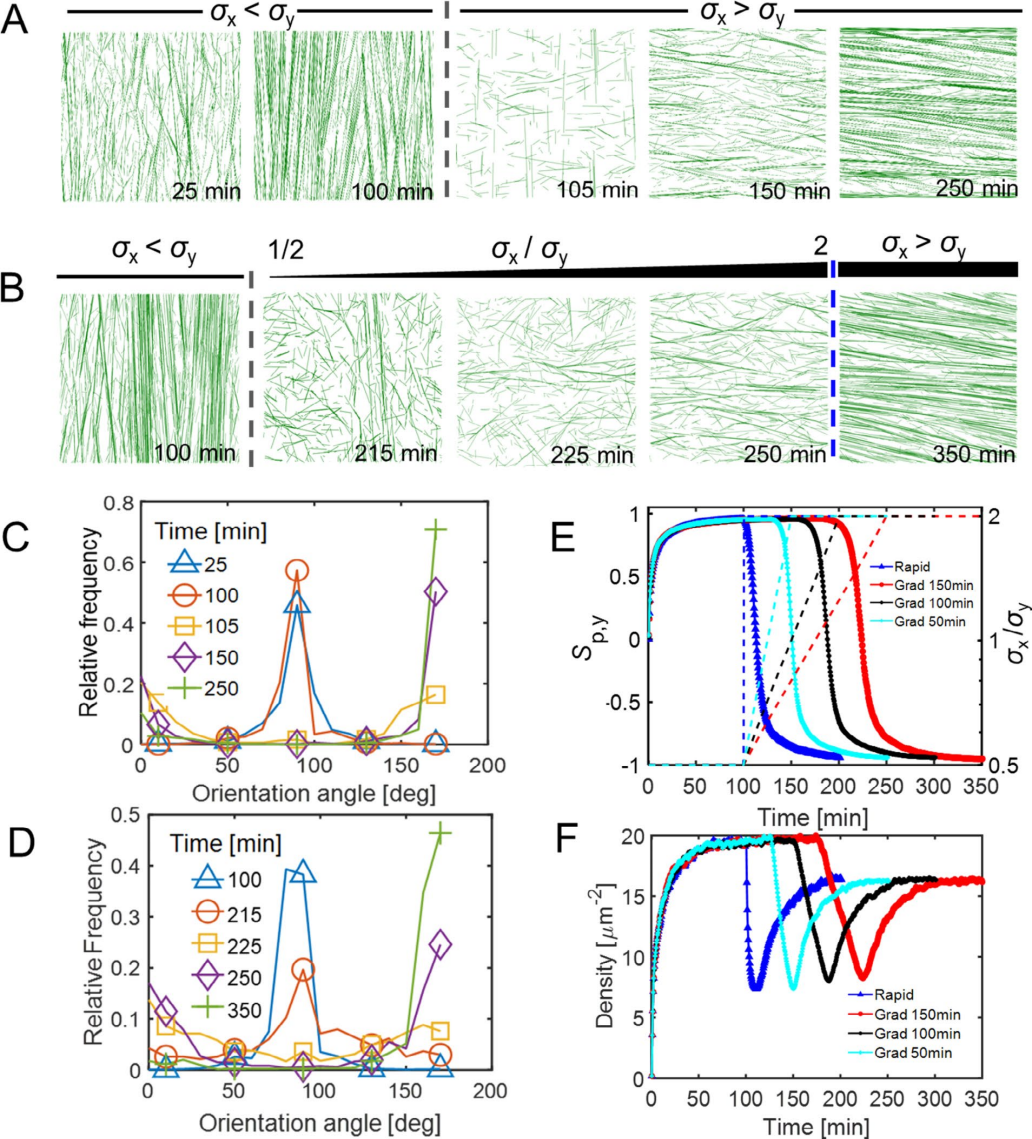
Dynamic stress pattern reorientation leads to remodeling of the microtubule network. (**A** and **B**) Time evolution of the MT network morphology (at various timepoints including 25, 100, 105, 150, 250 min for a rapid transition and 100, 215, 225, 250, 350 min for a gradual transition). Grey dashed line indicates the timepoint (100 min) after which reorientation of anisotropic stress takes place. The blue dash line indicates the timepoint after which anisotropic stress is stabilized. Initial network stress pattern is predominant in y direction. In **A**, stress pattern reorients instantaneously and becomes predominant in x direction after 100 min. In **B**, stress pattern gradually reorients until 250 min and becomes steady afterwards. (**C** and **D**) Relative frequency showing the distribution of microtubule orientation angle at various timepoints during stress pattern reorientation in A and B respectively. 90˚ is equivalent to the direction parallel to y axis, and 0˚ or 180˚ is equivalent to the direction parallel to x axis. A flat curve indicates homogeneous distribution of MT orientation angles. (**E**, **F**) Time evolution of the network order parameter *S*_p,y_ which is calculated with respect to the y direction (**E**) and network microtubule segment densities (**F**) in four cases: rapid stress transition/reorientation (blue dashed line), gradual transition of stress with reorientation within 150 min (red dashed line), 100 min (black dashed line), and 50 min (cyan dashed line). Cases represented by blue and red correspond to those shown in **A** and **B**, respectively. In **E**, the dashed lines represent time evolution of the value of stress anisotropy, *σ*_x_ / *σ*_y_, indicated by the right y axis.

When the principal direction of stress was rotated by 90˚ (from the y direction to the x direction) instantaneously at ~ 100 min, well-aligned microtubules were quickly depolymerized due to a reduction in their average lifetime compared to the value before 100 min (Fig. S6A). In addition, newly nucleated microtubules were polymerized longer in the new direction and then stabilized in the direction parallel to the new principal stress. Here, a change in the direction of microtubule alignment after the rotation of the principal direction of stress was evaluated by calculating the order parameter with respect to the y direction, *S*_p,y_. If microtubules are aligned well in the x direction after the rotation, *S*_p,y_ becomes close to -1. This is due to the abrupt change in the differentiated catastrophe frequency caused by the rotation of stress pattern. It took ~ 5 min for aligned microtubules to be transformed into randomly oriented microtubules. 50 min after the abrupt stress reorientation, most of microtubules were reoriented in the perpendicular direction (Fig. 2C), followed by stabilization and thickening of bundles due to a decrease in collision-induced catastrophe events.

When the stress pattern was gradually changed, microtubules were initially aligned in the y direction perpendicular to the new stress as in the case with the instantaneous stress change (Fig. 2D). As stress anisotropy (*σ*_x_ / *σ*_y_) was enhanced over time (150 min), more short microtubule segments emerged in the x direction. At ~ 215 min, the microtubule array became relatively homogeneous with randomly oriented microtubules (Fig. 2B, D). At 350 min, most of the microtubules were reoriented to the x direction, and bundles looked quite similar to those observed at the end of simulations run with the instantaneous stress change (Fig. 2A, B). The overall results were similar regardless of how long it took for the gradual stress change to take place. In the cases where it took 50 or 150 min instead of 100 min, *S*_p_ showed an increase and reached similar level at the end (Fig. 2E). The complete reorientation, in all cases, took ~ 50 min. In addition, it took ~ 20 min for the network to become homogeneous (i.e., *S*_p_ ~ 0) at a timepoint where stress became nearly isotropic (Fig. 2E). These values are comparable to a time range between 10 min and 2 h which is an experimentally determined rate for microtubule reorientation [56, 57]. The average lifetime of microtubules aligned in various directions did not show a significant difference between cases with instantaneous and gradual stress changes (Figs. S6A, B). Local heterogeneity of microtubule orientations was positively correlated with the stress pattern transition (Fig. S6C). Interestingly, during microtubule orientation, we noticed microtubule density was almost halved when the microtubule array became homogeneous, followed by a rescue when the new bundles formed (Fig. 2F).

### Stress gradients can generate fine-scale patterning of microtubule arrays

**Fig. 3:**
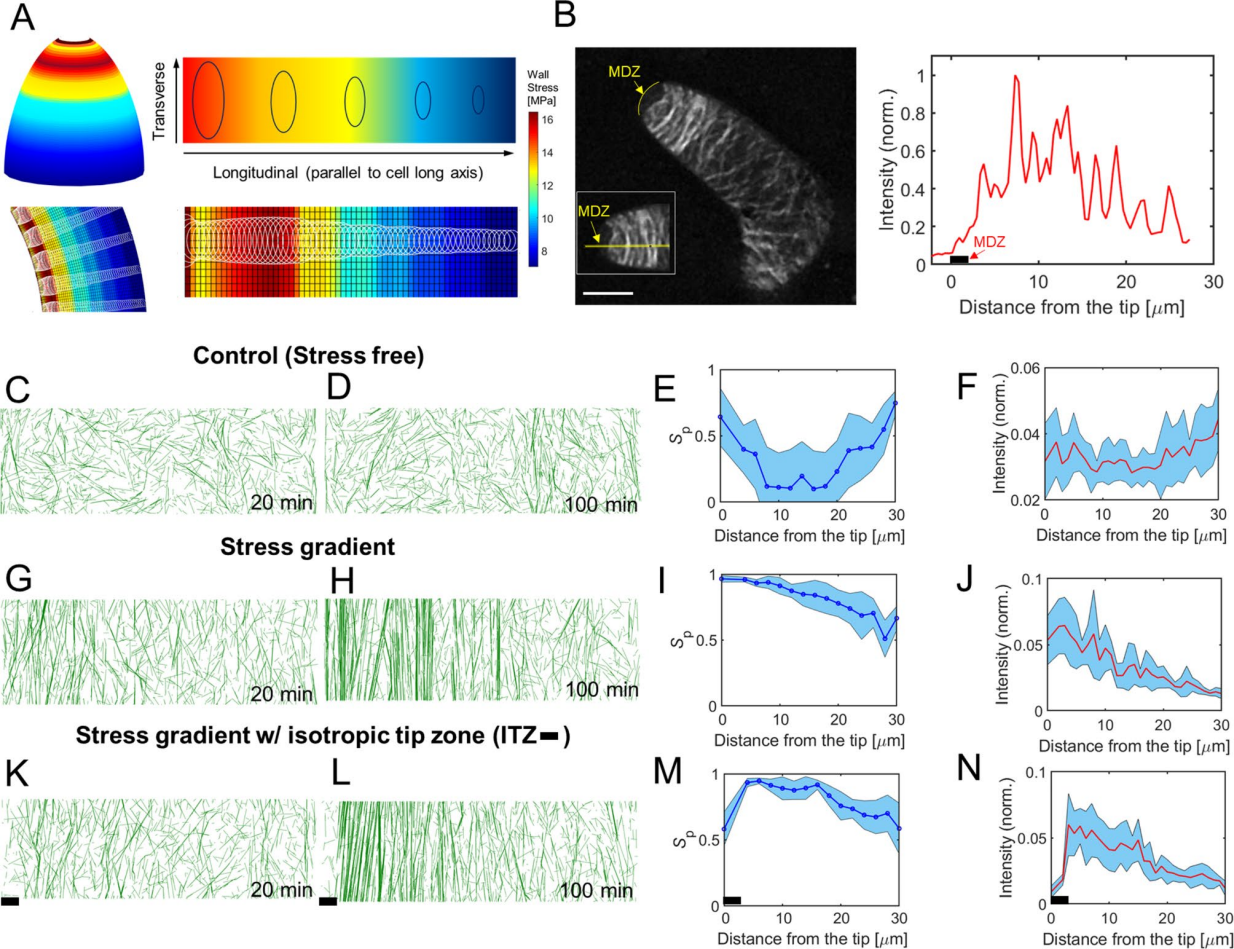
Physiological relevant stress gradient can induce distinguishable transverse microtubule bands. (**A**) Schematic of the stress pattern gradient pertinent to a finite element model of trichome morphogenesis. Magnitude of stress decreases from left to right, along the direction parallel to cell long axis while the stress is highly anisotropic and predominant in the transverse direction, indicated by the ellipses. The lower panels show an unfolded stress pattern based on the model results. The unfolded meshed stress pattern is mapped onto the simulation domain. (**B**) Representative confocal image showing microtubule localization in stage 4 young trichome branches (left panel). Transverse band is prominent near the cell apex. The MDZ is highlighted and marked in yellow. Scale bar: 10 μm. Right panel shows the intensity profile from the apex to the base in the image shown in the left panel. (**CN**) A catastrophe-inducing boundary condition is implemented on the two short edges of the simulation domain (30 μm x10 µm). Catastrophe-inducing boundary: when a growing microtubule encounters a boundary, it switches to a shrinkage state. The long edges are assumed to have periodic boundary conditions. MT band formation along the transverse direction is noticeable in cases with a stress gradient. (**C**, **G**, **K**) Early-time (20 min) and (**D**, **H**, **L**) steady-state (100 min) morphology of network in stress free (**C**, **D**), stress gradient (**G**, **H**), and stress gradient with isotropic stress in the tip zone (**K**, **L**, 2 μm tip zone marked by black bar). (**E**, **I**, **M**) Local order parameter of microtubules in subregions of cases in **D**, **H**, **L** respectively. For this, the network was divided into fifteen subregions, incrementally separated by 2 μm along the x direction. Blue curve indicates a mean value, and shaded region represents standard deviation (n = 10). (**F**, **J**, **N**) Normalized intensity of MT segments (tubulin dimers) as a function of the distance from the tip (left boundary), which is nearest to apex of a real cell. (**F**) For a homogenous network, the distribution of MT segments is relatively even along the cell long axis. (**J**) For a network with stress gradient, the distribution of MT segments peaks near the boundary and gradually decreases along the cell long axis. (**N**) With an isotropic stress zone near the tip (black bar), the distribution is similar to (**J**) except there is a decline in the intensity of microtubules in the zone. The distribution is consistent with the right panel in **B**

Based on known cell wall thickness gradient and a validated FE model [32], wall stress is non-uniform and anisotropic (Fig. 3A). The stress patterns in the FE model show principal stress increasing from the extreme apex to the apical flanks and shaft, then decreasing toward the base due to interactions between increasing cell radius and cell wall thickness toward the branch (Fig. 3A).

The initial focus was on cell flanks because microtubules in this region are highly aligned. The alignment, orientation, and density of microtubules in leaf trichoblasts have been extensively analyzed in Arabidopsis [30, 32, 58, 59], and similar complex cytoskeletal patterns are conserved in trichomes of several plant species [31, 33, 60]. The cell type is characterized by an obvious microtubule-depleted zone at the extreme apex and a highly aligned transverse collar of microtubule array that spans from the apex shoulder to a more distal region of the shaft. The microtubule-depleted zone generates a patch with isotropic cell wall and this material property gradient mediates cell tapering via a polarized diffuse growth mechanism [32, 33]. A representative microtubule localization is shown in Fig. 3B. This transverse arrangement is required to pattern the synthesis of highly anisotropic cellulose fibers in wall. Small deviations from the transverse orientation of fibers lead to cell swelling and aberrant cell morphologies in Arabidopsis leaf hairs and cotton fibers [31–33, 61, 62].

To generate a realistic gradient of stress magnitude from the cell flanks and shaft, we projected the stress pattern of the curved FE model of the branch to a rectangular simulation space of 30 × 10 μm (Fig. 3A). To analyze boundary effects, we imposed four different types of conditions on two boundaries normal to the longitudinal cell orientation: catastrophe-inducing, reflective, repulsive, and a periodic boundary as a control (Fig. S7A). The periodic boundary condition was not employed in the presence of the stress gradient to prevent microtubules from crossing the boundaries and thus experiencing an abrupt change in the stress pattern. We first tested cases (i) without the stress pattern in the presence of the periodic boundary, (ii) without stress with the catastrophe-inducing boundary condition, and (iii) with global isotropic stress. Under the stress-free condition with the periodic boundary condition in all directions, the microtubule array was homogeneous with small bundles, leading to an insignificant variation in the local order parameter, *S*_p_(*z*), and local density, *I*(*z*), of microtubules in the z direction parallel to the longitudinal axis of the cell (Figs. S8A, B). Note that z = 0 corresponds to the location of the left boundary. When two catastrophe-inducing boundaries were imposed without stress, more aligned bundles aggregated near regions close to the two boundaries, characterized by an increase in *S*_p_(*z*) and *I*(*z*) (Fig. 3 C-F and S8A, B, Movie S1). Under the isotropic stress condition without stress, *S*_p_(*z*) showed a qualitatively similar distribution. However, isotropic stress significantly enhanced *S*_p_(*z*) globally by ~ 0.3 due to increased average length and lifetime of microtubules (Table 1). These results indicate that catastrophe-inducing boundary alone could only locally increase microtubule alignment. As a next step, we implemented a stress gradient. We observed clustering of microtubules at the tip (i.e., left boundary), regardless of the type of imposed boundary conditions (Fig. S7F). With catastrophe-inducing, reflective, and repulsive boundaries, bundles were formed in the direction parallel to principal stress (i.e., perpendicular to the cell axis) in regions with large stress (Fig. 3G-J, S7B-E, Movie S2). *S*_p_(*z*) showed a similar tendency to that in the control case (Fig. 3E, I). Regions with higher stress showed *S*_p_(*z*) greater than 0.6 within 20 μm from the distal boundary. By contrast, in regions proximal to the base region with lower stress, microtubules were noticeably less dense and more homogenous in all cases. This is attributed to a fraction of disordered microtubules that turn over quickly due to catastrophe induced by frequent collisions between microtubules. These simulations reflect the appearance of microtubules with diminished density near the branch base (Fig. 3B).

The collar necessarily includes the MDZ. Microtubules at the MDZ boundary function as diffusion barrier for signaling complexes that form actin meshworks at the cell apex [63], and generate a sharp material property gradient due to the isotropic tip and the highly anisotropic flank and shaft [32, 33]. Therefore, these conditions reflect the stress distributions of a real cell, and contain known spatial gradients in stress anisotropy. To test whether in vivo stress patterns at the cell apex could generate MDZ, we incorporated a small apical zone of 2 μm in length with isotropic stress (*σ*_x_ / *σ*_y_ = 1) at the left boundary (Fig. 3B and K-N, Movie S3). Note that the isotropic stress is also present in the FE model of apex (Fig. S9), and isotropic stress is a general feature of hemispherical domes [33]. The reported value of stress anisotropy in the flank region of a trichome was also around 2:1 (Fig. S9). Interestingly, the apical isotropic stress zone resulted in a very localized sparse, disordered population of short microtubules similar to the base (Fig. 3K-N), with a sharp decrease in *S*_p_(*z*) near the tip zone. The rest of the domain showed a similar pattern to that observed with an intact stress gradient. The relative intensity of microtubules showed consistent results with microtubule morphology. Thus, the combination of spatially varying gradients of stress magnitude and anisotropy could generate a microtubule pattern that closely resembles the cortical array observed in living cells.

## Discussion

The microtubule-microfibril coalignment module of gene functions integrates an evolutionarily conserved cytoskeletal protein with cell wall synthesizing protein complexes that generate a viscoelastic anisotropic cell wall [5, 9]. Spatial and temporal control of these activities across wide spatial scales have enabled morphological diversification in the plant kingdom [2, 6, 64]. Stress patterns, which reflect both geometric and compositional features of plant tissues and cells, have the potential to provide a robust mechanism for the cortical microtubule system to “sense” and respond to geometry and composition indirectly [15, 22, 26, 43, 57]. The mechanism by which microtubules might sense cell wall stress is not known.

In this study, we analyzed how coupling between wall stress and microtubule dynamics can potently orient the cortical array and reconstitute patterns that arise from basic geometric and compositional properties of plants cells. Specifically, we developed a computational framework to predict the organization of microtubule arrays with an assumption that microtubules can sense the magnitude and direction of tensile forces in the cell wall; it was assumed that direction-dependent stress affects one of the stochastic behaviors that the plus end of microtubules exhibits (Table 2). Our simulations recapitulated physiologically relevant alignment and reorientation of microtubules that occur in cells and indicate that wall stress can align the cortical microtubule array as a function of cell geometry. Specifically, we showed that anisotropic stress can align microtubules (Fig. 1A, B, D) and decrease the time required for the array to reach a steady state (Fig. 1E). We identified the most efficient stress-sensitive parameter that can lead to more conspicuous microtubule alignment. A stress-dependent variation in the polymerization rate and catastrophe frequency was the most potent means to rapidly establish a microtubule array aligned with anisotropic stress. Note that our assumption of an increased polymerization rate or a decreased catastrophe frequency due to increasing stress is consistent with results from in vitro studies that probed effects of tensile forces on the plus-end dynamics of microtubules [50, 51]. The plus ends of microtubules switch between growth, pause, and shrinkage states with dynamic instability, so a large fraction of short microtubules can disappear very easily. If stress increases the polymerization rate or reduces the catastrophe frequency of microtubules oriented in a certain direction, the aligned subpopulation of short new microtubules can grow fast by making polymerization more dominant. Suppression of the catastrophe events can break symmetry between polymerization and depolymerization more so that microtubules are aligned faster and to a greater extent.

In growing cells and tissues, the wall stress fields are constantly remodeled (albeit slowly) as the geometry and wall material properties change. Wall stress and microtubule alignment can also change as a function of the growth dynamics of underlying tissues [42]. We conducted the microtubule array simulations with time-varying stress patterns to evaluate the robustness of the system (Fig. 2). We found that more stable states in the microtubule simulations were not predetermined or fixed outcomes defined by the initial input variables; microtubules were reoriented in response to a change in the stress patterns. We observed that during microtubule reorientation, bundles with different orientations could coexist with rather homogeneous morphology. This was previously observed in experiments where mechanical perturbation was employed to alter microtubule patterns [22, 44].

Cell wall stresses serve as useful multiscale patterning elements to modulate microtubule arrays because the stresses are non-uniform on a cell surface and depend on both geometric and cell wall compositions. For example, stresses are locally concentrated and aligned at the interface where unpaired outer periclinal walls pull upward on anticlinal walls, and microtubules at this location are highly coaligned [15, 27]. The magnitude of cell wall stress is also inversely proportional to cell autonomous parameters like wall thickness. In isolated leaf trichoblasts, subcellular cell wall thickness gradients exist [32], implying the existence of stress gradients along the cell wall. We showed that the spatial information of the direction and magnitude of stress can be decoded to generate an ordered microtubule array with a tip to base density gradient that mirrors that of living cells (Fig. 3B, G-J).

Living trichoblasts have an apical MDZ that may reflect an additional gradient in stress anisotropy. In domed, cylindrically-shaped trichoblasts, root hairs, pollen tubes, and moss protonema, stress will be more isotropic at the apex than the cell flanks. Based on geometry alone, the flanks of cylindrically-shaped cells are expected to have anisotropy ratios of ~ 2:1. This value exceeds the threshold of 1.5:1 that was needed to order the microtubule network in simulations (Fig. S4). The apex dome is an important site for signal transduction and cytoskeletal patterning [63], and it has been hypothesized that ROP-dependent recruitment of catastrophe-inducing Kinesin13-related proteins was involved in maintaining the MDZ [59]. However, our computational simulations showed that a combination of spatially defined gradients of stress anisotropy and magnitude can pattern a microtubule array with the basic alignment and density features that reflect the MDZ and the apical collar of cortical microtubules (Fig. 3B, K-N). We probed the effects of periodic or catastrophe-inducing boundaries on microtubule alignment with isotropic stress or without stress. We observed that microtubule density and alignment patterns were not similar to the experimental observations if anisotropic stress is not present (Fig. S8 and Supplementary Text). These results are important because they suggest that stress-dependent alignment of the microtubule array is a plausible and sufficient feedback control mechanism to persistently integrate cell geometry and the microtubule-microfibril systems during morphogenesis. A similar control mechanism may operate in the apical dome of cotton fibers [33]. Dominant aligned stresses at the shoulder of the dome can generate a population of aligned microtubules. These persistent and aligned microtubules can serve as a catastrophe-inducing boundary when microtubules that originate from the apex encounter them at a steep angle.

**Fig. 4:**
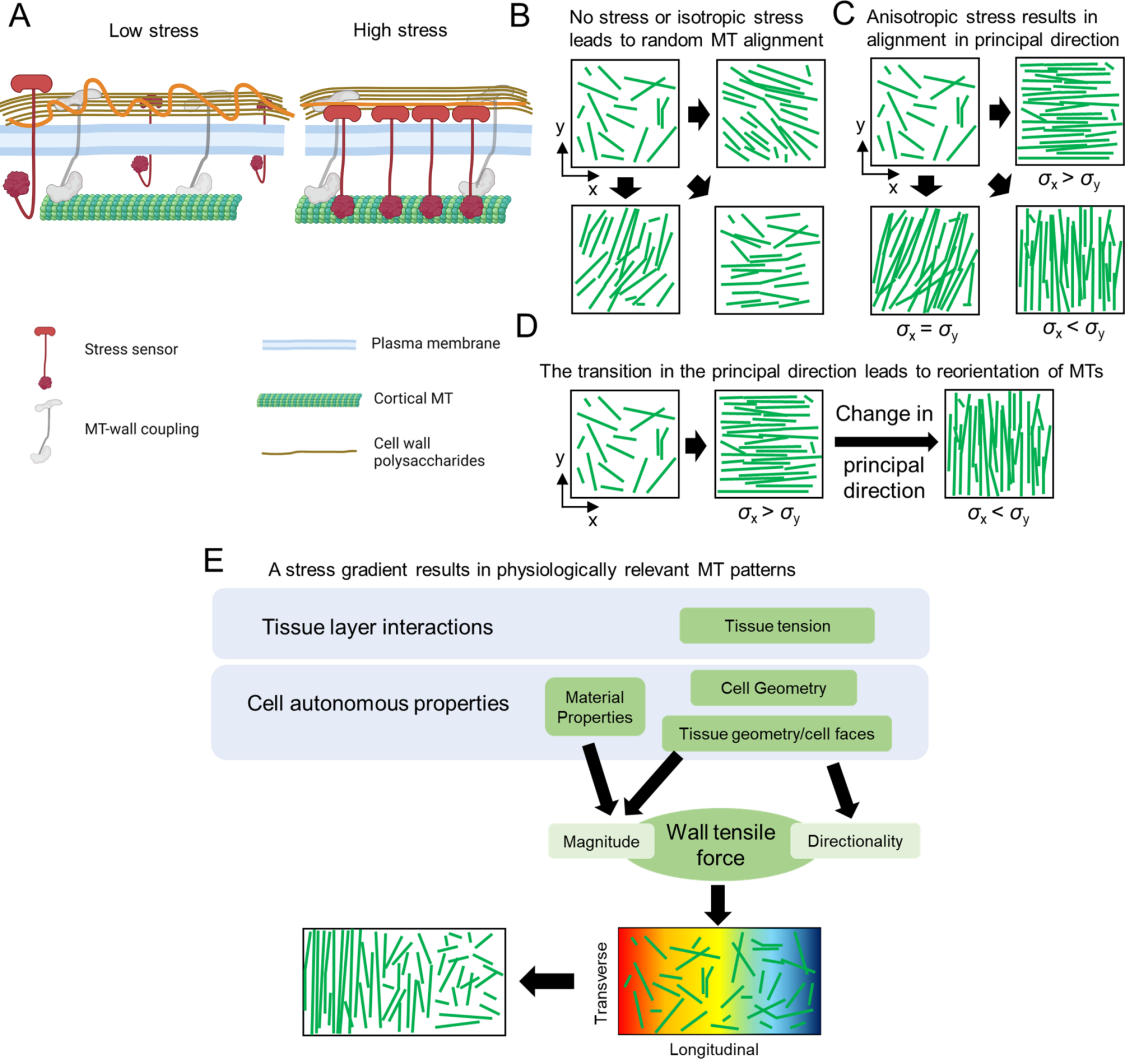
A proposed model for how mechanical intervention biases the microtubule network and leads to its global reorientation. (**A**) Proposed model for how potential stress sensors act on a pre-structured microtubule-cell wall system in the case of low stress (left panel) and high stress (right panel). The schematic is created with BioRender. (**B**) When MTs are not influenced by mechanical stress, the network can form aligned MT bundles in random directions. (**C**) As the network is subject to anisotropic stress pattern, MTs can align and orient in the direction parallel to the principal stress, to various extents based on which parameter is regulated by stress. (**D**) Time-dependent transition of the principal direction of network stress can lead to dynamic remodeling and MTs thus globally reorient the MTs. (**E**) With a biological cell-based stress gradient, MTs can form physiologically relevant patterns with transverse bands near the cell apex during anisotropic diffuse growth, reminiscent of the alignment of MTs parallel to tip-biased, shape-derived, material-property-based anisotropic cell wall stress

The mechanism by which wall stress modulates the microtubule cytoskeleton is not known. The microtubule-severing protein, katanin, is unlikely to be the sensor because it is required to propagate super-cellular stress patterns across cell boundaries but does not affect the ability of cells to sense stress [57]. Our simulations suggest that the sensor responds to both the magnitude and direction of the force, so it would sense stress at two or more points along a line. Therefore, it is not likely that the localized activation of stress-gated ion channels could generate such a highly directional response. Microtubules do not display diffusive motions regardless of their orientations [11], implying that each microtubule is coupled, either directly or indirectly, to an immobile wall component (or membrane environment) in a stress-independent manner. The stress sensor might operate in concert with or independently of this constitutive coupling factor (Fig. 4A). Microtubule alignment in response to stress occurs throughout the cell surface and is independent of their growth status, suggesting that the ability to sense stress is widely distributed and linked to some sort of load-bearing polysaccharide networks. Growth-associated wall strain in trichoblasts [32] and the anticlinal walls of epidermal pavement cells [15] are highly anisotropic and maximal in a direction orthogonal to a major wall stress direction, further indicating that the sensor would sense stress rather than strain that occurs due to irreversible cell growth.

A plausible mechanistic model for the stress sensor was proposed by Williamson more than 30 years ago [45]. Based on the directional nature of the microtubule response to stress, he suggested the existence of integral membrane receptors with an extracellular stress-sensing domain and a cytoplasmic microtubule coupling domain. He proposed that stress sensing would occur via load-dependent interactions with newly synthesized amorphous cellulose chains that would undergo large conformational changes in response to stress. We now know that load-bearing crystalline cellulose microfibril networks [5] and presumably other polysaccharides that are strongly associated with cellulose [5] can change conformation as a function of stress magnitude and direction. These receptors could display a catch-bond behavior for a binding site that is generated by high stress, meaning they would exhibit a decrease in the dissociation rate for wall binding in the presence a higher tensile force [65]. Engagement of the catch bond in the extracellular domain could be coupled with a change in the activity of the microtubule coupling domain. Over time, the receptors would accumulate in a directional manner along the load-bearing network and physically interact with microtubules or other microtubule-associated proteins that increase the longevity of aligned microtubules. When stress dissipates, the molecular complexes could disassemble for a later round of use. Future efforts will be needed with a focus on finding such a receptor.

## Conclusions

In this paper, we demonstrated how multiple aspects of wall stress can provide patterning information to orient the microtubule array at cellular scales (Fig. 4B-D). Additional research is needed to experimentally analyze dynamic properties of microtubules as they experience changing stress. We predict that plus-end dynamics is a key point of regulation. We expect this patterning system operates to link any process that changes the stress distributions. This includes tissue-layer interactions that can generate strong stresses in the epidermis, as well as geometric and compositional variables that have cell-autonomous effects on cell wall stress (Fig. 4E). These multiscale cellular interactions can affect the magnitude, direction, and anisotropy of stress, and these parameters can operate alone or in combination to influence the microtubule array. We predict that this regulatory scheme operates in all plant cells that use a polarized diffuse growth mechanism. This feedback control system would not only enable cells to maintain cell wall integrity by generating oriented fibers in response to high stress, but also enable the cytoplasm to sense cell geometry and program predictable growth outputs.

## Methods

### Microtubule dynamics

**Fig. 5:**
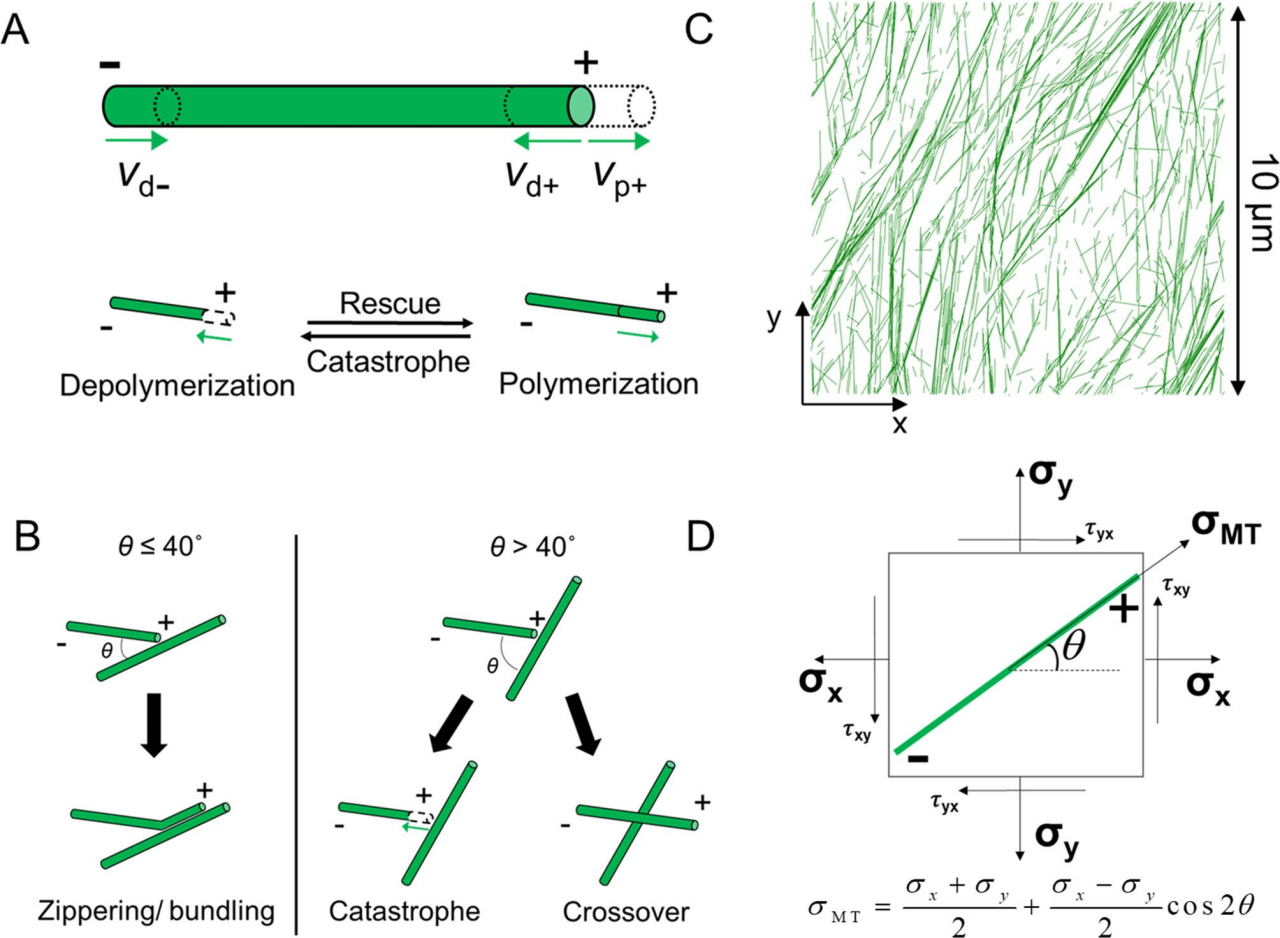
Model schematic and microtubule dynamics. (**A** and **B**) Schematic of MT dynamics, including stochastic events and angle-dependent collision/deterministic events. (**A**) Stochastic events include polymerization (v_p+_) and depolymerization (v_d+_) at the plus end and constant slow depolymerization (v_d−_) at the minus end, rescue, and catastrophe. (**B**) Angle-dependent collision-based events of MTs include zippering at a shallow angle (< 40º) and catastrophe or crossover beyond a critical angle (> 40º). (**C**) MT simulation domain is a 10 × 10 μm (initial domain size) square network with periodic boundary conditions in both x and y directions. MTs in the simulation domain are composed of serially connected small segments representing tubulin dimers, as shown in green. (**D**) Mathematical relationship between local principal stress and stress acting on individual microtubules depending on their angle of orientation

Each microtubule is represented by serially connected segments with 100 nm in length. The nucleation of new microtubules takes place at the rate of 10 μm^− 2^·min^− 1^ at a random location without dependence on existing microtubules. Following approaches used in a previous study [66], it is assumed that the dynamic behavior of the plus end of microtubules can switch between growth, shrinkage, and pause states (three-state dynamics), whereas their minus end always undergoes slow shrinkage at a constant rate (Fig. 5A).

Based on experimental observations, it is assumed that a collision between two microtubules with a contact angle smaller than 40˚ results in zippering, meaning that one of the microtubules changes its orientation to align with the other microtubule (Fig. 5B). A spacing between microtubules is set to 25–50 nm which is close to observations in animal and plant cells [67, 68]. A collision with a contact angle greater than 40˚ leads to either crossover or catastrophe. The probability of catastrophe after a collision is set to a value between 0.2 and 0.8 [66]. Stochastic dynamic events of microtubules were updated once per 0.001 min, and collisions between microtubules were considered once per 0.05 min (Fig. S1C), which is consistent with the previous model [66]. For this study, we assumed an unlimited pool of free tubulin dimers.

The parameters used to depict the stochastic properties of microtubules and deterministic behaviors of microtubule-microtubule interactions are listed in Table 2 and S1 (Supplemental Text).

**Table 2:** List of dynamic parameters of microtubules for three-state models

Parameter	Description	Value [11]
*f*_*gp*_	Frequency of a transition from growth to pause	0.47 min^− 1^
*f*_*gs*_	Frequency of a transition from growth to shrinkage	0.97 min^− 1^
*f*_*pg*_	Frequency of a transition from pause to growth	0.51 min^− 1^
*f*_*ps*_	Frequency of a transition from pause to shrinkage	0.24 min^− 1^
*f*_*sg*_	Frequency of a transition from shrinkage to growth	0.87 min^− 1^
*f*_*sp*_	Frequency of a transition from shrinkage to pause	0.23 min^− 1^
*v* ^*p*^_*g*_	Growth rate (+ end)	3.69 μm/min
*v* ^*p*^_*s*_	Shrinkage rate (+ end)	5.80 μm/min
*v* ^*m*^_*s*_	Shrinkage rate (- end)	0.53^*^ µm/min

*: 0.253 (2.78 μm/min) + 0.084 (− 1.96 μm/min) + 0.663 (0) Weighted average from [11]. Minus end growth phase (25.3%), pause phase (66.3%) and shrinkage phase (8.4%) considered in the weighted average.

### Stress pattern and computational domain

To analyze the effects of local cell wall stress on microtubules, realistic stress patterns from validated FE models [32] were spatially discretized onto the mesh elements in a simulation space with various geometries. For the square domain, we used 10 × 10 μm for its dimension with the periodic boundary condition (PBC) in the x and y directions (Fig. 5C). We confirmed that this domain size is large enough to avoid finite-size effects by comparing results to those obtained with a larger domain with 20 × 20 μm in size (Figs. S2A, B). Stress patterns mapped onto the square matrix undergo either no, sudden, or gradual change during simulations. For the stress pattern with no change, stress components remain constant during the entire duration of simulations (100–200 min). For the stress pattern with a sudden change, an initial stress pattern is maintained for ~ 100 min, and then a predominant stress direction is rotated by 90˚ and maintained till the end. For the stress pattern with a gradual change, stress components are varied linearly; the stress pattern is initially predominant in y direction, and the y component of stress is reduced linearly over time, whereas the x component is linearly increased. The x and y components of the stress become identical at the middle of these linear changes. For example, if it takes 50 min for the linear changes, stress becomes isotropic at 25 min. At the end, the extent of the stress anisotropy becomes identical to that of the initial stress anisotropy. It takes 50–150 min to complete the linear changes in the stress pattern, which is comparable to the timescales of tens of minutes or a few hours required for the pattern of cortical microtubules to be reoriented [69, 70]. This similarity was mostly attributed to our assumption that microtubule dynamics changes instantaneously with a stress pattern transition. However, the time scale for mechanical stimuli changes does significantly vary, depending on specific cell and tissue types. The microtubule reorientation could be delayed by up to 7 h after mechanical perturbations [43]. To address this, we would consider implementing a time delay between the microtubule response and the stress anisotropy change in our future work. This would require experimental validation of how stress could propagate through the network. Upon establishment of such understanding, we could further compare the simulation results with various time delays for microtubule responses in vivo.

For the rectangular domain, we used 30 × 10 μm for its dimensions in the x and y directions, respectively. It is assumed that the longer direction (*x*) is parallel to the cell long axis, and the shorter direction (*y*) represents the transverse direction. In the stress pattern mapped onto the domain, the extent of stress anisotropy is relatively constant along the cell long axis, whereas the magnitudes of stresses decrease from left (the cell apex) to right (the cell base) to impose a stress gradient. The PBC is applied in the y direction to avoid finite-size effects. In the x direction, unless specified, there is no PBC to avoid an abrupt change in the stress across the boundaries. Instead, three different types of boundary conditions are imposed in the x direction; the catastrophe-inducing boundary enforces growing microtubules to switch to a shrinkage state. The reflective boundary causes microtubules growing toward the boundary to change the direction with a new reflective angle equal to the incident angle. The repulsive boundary pauses a growing microtubule as soon as the microtubule makes contact to the boundary. These types of boundary conditions have been used in previous modeling studies [34, 66]. All simulation were developed and performed by MATLAB R2020a via a supercomputing cluster, Bell, operated by Information Technology at Purdue (ITaP) at Purdue University.

### A constitutive relationship between local stress and microtubule dynamics

The constitutive relationship between local stress and microtubules dynamics was devised from recent experiments showing that a tensile force up to ~ 10 pN increases the growth rate and the rescue frequency but decreases the depolymerization rate and the catastrophe frequency [50]. Based on this observation, it is assumed that these four dynamic parameters in our model exhibit mechano-sensitive behaviors. It is assumed that the magnitude of local stress mapped onto a microtubule in a certain orientation affects one of the four dynamic parameters on the plus end of the microtubule (Fig. 5D). Using the stress transformation equation in continuum mechanics, we calculate the orientation-dependent local stress, *σ*_MT_ :

1 $${\sigma _{{\text{MT}}}}=\frac{{{\sigma _x}+{\sigma _y}}}{2}+\frac{{{\sigma _x} - {\sigma _y}}}{2}\cos 2\theta$$

where *σ*_*x*_ and *σ*_*y*_ are normal stresses, and *θ* is the orientation of microtubules measured relative to the + x direction in the simulation space. Note that shear stress is assumed to be negligible in this equation, based on predictions from a previous FE model [32]. A constitutive relationship is defined between *σ*_MT_ and either of the growth rate, the shrinkage rate, the catastrophe frequency, or the rescue frequency (Figs. S1A, B). We assume a linear form for the constitutive relationship. Note that relationships between tensile force and the four dynamic parameters observed in experiments were close to exponential functions [50].

### Quantification of the order parameter and time constants

The order parameter (*S*_p_) is calculated to evaluate the extent of ordering of microtubules:

2 $${S_{\text{p}}}=\frac{{\sum\limits_{{i=1}}^{{{N_{{\text{MT}}}}}} {{l_i}[{{\cos }^2}({\theta _i} - {\theta _p}) - } {{\sin }^2}({\theta _i} - {\theta _p})]}}{{\sum\limits_{{i=1}}^{{{N_{{\text{MT}}}}}} {{l_i}} }}$$

where *N*_MT_ is the total number of microtubules, *θ*_*p*_ is a reference direction, and *θ*_*i*_ and *l*_*i*_ are the angle and length of each microtubule segment, respectively. *θ*_p_ is different depending on cases, as explained in each case in the results section. We quantify a time constant to indicate the rate of a change in the order parameter. The first time constant (*τ*_p_) represents time at which *S*_p_ reaches half of its steady-state value. The second (*τ*_*p*,2_) and third (*τ*_*p*,3_) time constants indicate time at which *S*_p_ reaches 75% and 87.5% of the steady-state value, respectively. In addition, we also calculate the instantaneous rate of a change in *S*_p_.

### Live cell imaging

We performed live cell imaging for stage 4 trichome in which 2–3 short, blunt, branches were present. *Arabidopsis thaliana* seedlings were grown in Murashige and Skoog medium under continuous illumination. Single channel images were performed and collected with mCherry:MBD seedlings that have been previously described [63]. Arabidopsis trichomes were imaged at 10–12 DAG, where confocal fluorescence microscopy was performed using a Yokogawa spinning disk CSU-X1 head mounted on a Zeiss Observer.Z1 inverted microscope. Stages 2–4 trichomes were imaged using a 100X PlanApo 1.46 oil-immersion objective while mCherry was excited by 561 nm laser lines. Minute-scale images were acquired by Evolve 52 camera (Photometrics) through band-pass filters (482.35 and 617/73; Semrock). The collected z-stacks were analyzed in ImageJ.

## Electronic supplementary material

Below is the link to the electronic supplementary material.

Supplementary Material 1

Supplementary Material 2

Supplementary Material 3

Supplementary Material 4

## Data Availability

The datasets used and/or analyzed during the current study are available from the corresponding author on reasonable request.

## List of abbreviations

CESA: cellulose synthase
MDZ: microtubule depletion zone
2D: two-dimensional
3D: three-dimensional
MT: microtubule
PBC: periodic boundary condition
FE: finite element
