# Supplementary material for: Probing stress-regulated ordering of the plant cortical microtubule array via a computational approach
Source: BMC Plant Biol. 2023 Jun 9;23:308. doi: 10.1186/s12870-023-04252-5 (PMC10251582; doi:10.1186/s12870-023-04252-5)
Supplement: Supplementary file 1 — Supplementary Material 1 [file 12870_2023_4252_MOESM1_ESM.pdf]

SUPPLEMENTAL INFORMATION

Probing stress-regulated ordering of the plant cortical microtubule array via a computational approach

Jing Li

Weldon School of Biomedical Engineering, Purdue University

206 S Martin Jischke Dr, West Lafayette, IN 47907

Daniel Szymanski*

Botany and Plant Pathology, Purdue University

915 West State Street, West Lafayette, IN 47907

Taeyoon Kim*

Weldon School of Biomedical Engineering, Purdue University

206 S Martin Jischke Dr, West Lafayette, IN 47907

***** Corresponding authors, email: kimty@purdue.edu and szymandb@purdue.edu

Stress anisotropy and collision-induced catastrophe coregulate microtubule ordering

We showed earlier that microtubule can form bundles without stress anisotropy (Fig. S3C). Such stress-free alignment is mainly attributed to collision-induced catastrophe. Although zippering, the alignment of two microtubules after collision with small contact angles, can help microtubules align, the reorientation of microtubule bundles can be facilitated only by the collision-induced catastrophe. Microtubules can turn over more frequently in the presence of the collision-induced catastrophe, which helps microtubules align with each other. The collision-induced catastrophe could be more or less important if there is stress anisotropy that can also lead to microtubule ordering. To understand how these two different mechanisms facilitate microtubule alignment in a cooperative or antagonistic manner, we ran simulations with different probabilities for the collision-induced catastrophe and different levels of stress anisotropy. The probability of the catastrophe (P_{cat}) was varied between 0.2 and 0.8 to be consistent with the previous literature [1]. In cases with a stress-dependent variation in three parameters – the polymerization rate, the depolymerization rate, and the rescue frequency – the low probability of collision-induced catastrophe resulted in nearly randomly oriented microtubules due to substantial increases in cross-over events. As P_{cat} increases, the dependence of microtubule ordering on the degree of stress anisotropy became stronger in cases with all the three parameters (polymerization, depolymerization rates and rescue frequency) varied by stress (Fig. S5). The cases with the polymerization rate and the rescue frequency as a stress-sensitive parameter showed almost equal dependence of microtubule ordering on P_{cat} and stress anisotropy level (Figs. S5A, D). By contrast, the case with the depolymerization rate varied by stress showed that microtubule ordering is more sensitivity to P_{cat} than stress anisotropy (Fig. S5B). At small stress anisotropy level, the order parameter in the case with the depolymerization rate varied by stress significantly increased when

P_{cat} was changed from 0.6 to 0.8. However, it remained at very small values when P_{cat} was below this range, unlike order parameter in the cases of the polymerization rate and the rescue frequency varied by stress showing a consistent increase with an increase in P_{cat}.

In the case with the catastrophe frequency modulated by stress, microtubules were aligned well even at low P_{cat} when intermediate stress anisotropy level was imposed (Fig. S5C). Both stochastic catastrophe and collision-induced catastrophe contribute to the turnover of microtubules. By decreasing the stochastic catastrophe frequency, microtubule ordering became less dependent on stress anisotropy level since the turnover of microtubules are predominantly regulated by collision-induced catastrophe. By contrast, when stochastic catastrophe events take place frequently, microtubule ordering depends more on stress anisotropy because microtubule turnover became more dependent on free catastrophe (Figs. S5E-F).

In sum, the combinative effects of stress anisotropy and collision-induced catastrophe result in microtubule alignment and ordering. Stress anisotropy directly impacts the efficiency of the alignment of microtubules with the direction of principal stress, whereas collision-induced catastrophe controls the portion and turnover of misaligned microtubules.

Table S1. Stochastic and deterministic event parameters in addition to dynamic instability.

Notation	Value	Description
θ_Z	40˚ [2, 3]	Entrainment angle upon collision
f_{nuc}	100 [μm^{-2} min^{-1}]	Nucleation rate
p_{cat}	0.2 - 0.8	Probability of catastrophe upon collision
p_{zip}	1.0 [3]	Probability of zippering
δ	0 – 50 nm	Spacing between bundled MTs
p_g	0.65 [4]	Phase of growth %
p_p	0.10 [4]	Phase of pause %
p_s	0.25 [4]	Phase of shorten %
δ_{seg}	0.1 [μm]	MT segment length

Supplemental References

1. Ambrose, JC, Wasteneys, GO. CLASP modulates microtubule-cortex interaction during self-organization of acentrosomal microtubules. Mol Biol Cell. 2008;19(11):4730-7.

2. Dixit, R, Cyr, R. Encounters between dynamic cortical microtubules promote ordering of the cortical array through angle-dependent modifications of microtubule behavior. Plant Cell. 2004;16(12):3274-84.

3. Shaw, SL, Kamyar, R, Ehrhardt, DW. Sustained microtubule treadmilling in Arabidopsis cortical arrays. Science. 2003;300(5626):1715-8.

4. Allard, JF, Wasteneys, GO, Cytrynbaum, EN. Mechanisms of self-organization of cortical microtubules in plants revealed by computational simulations. Mol Biol Cell. 2010;21(2):278-86.

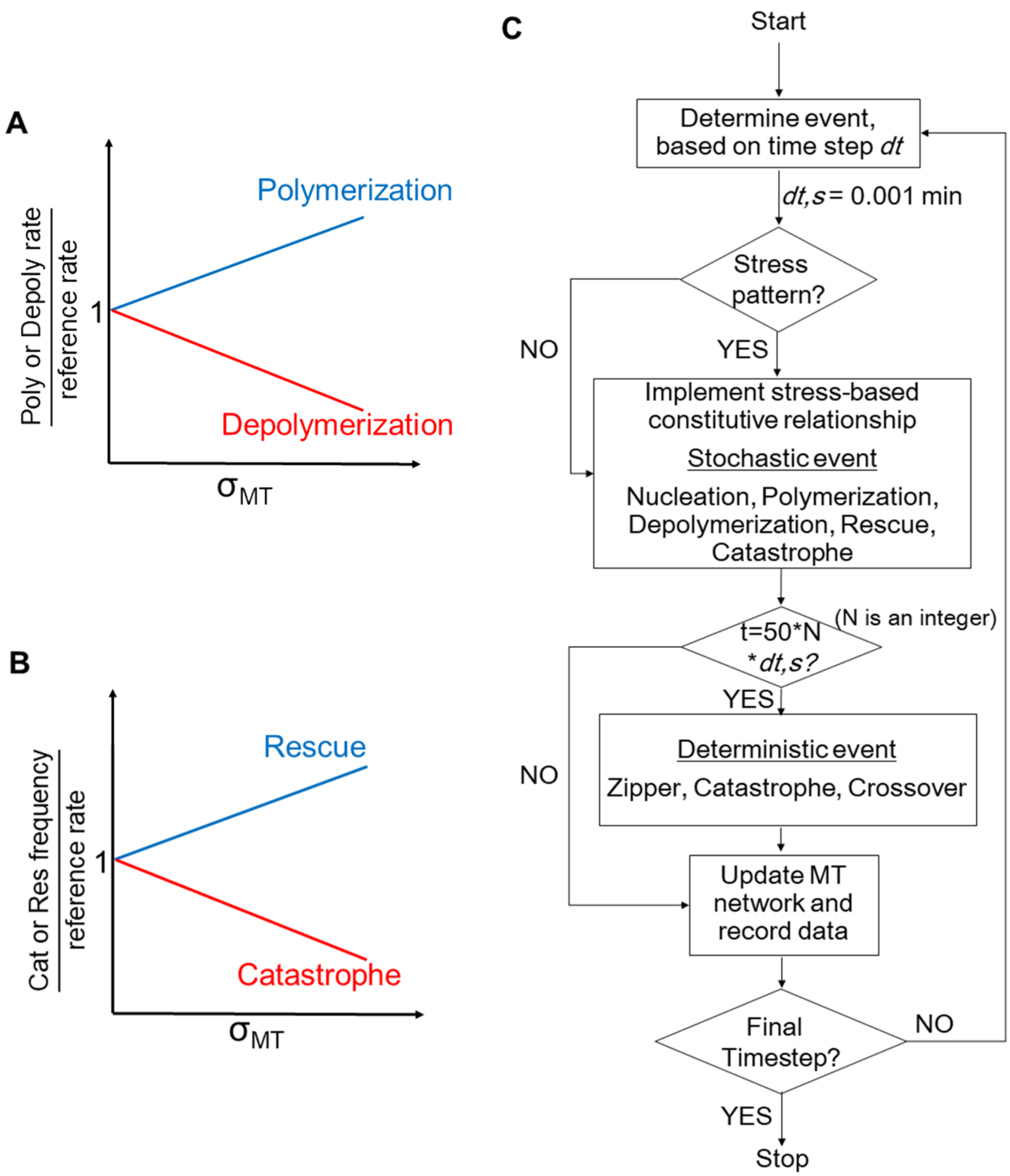

Figure S1. Model description in details. (A-B) The constitutive linear relationship between plus-end dynamics and the stress acting on microtubules. (A) An increase in stress enhances the polymerization rate and suppresses the depolymerization rate, following the linear relationship. (B) An increase in stress enhances the rescue frequency and suppresses the catastrophe frequency, following the linear relationship. (C) The main flowchart showing simulation steps.

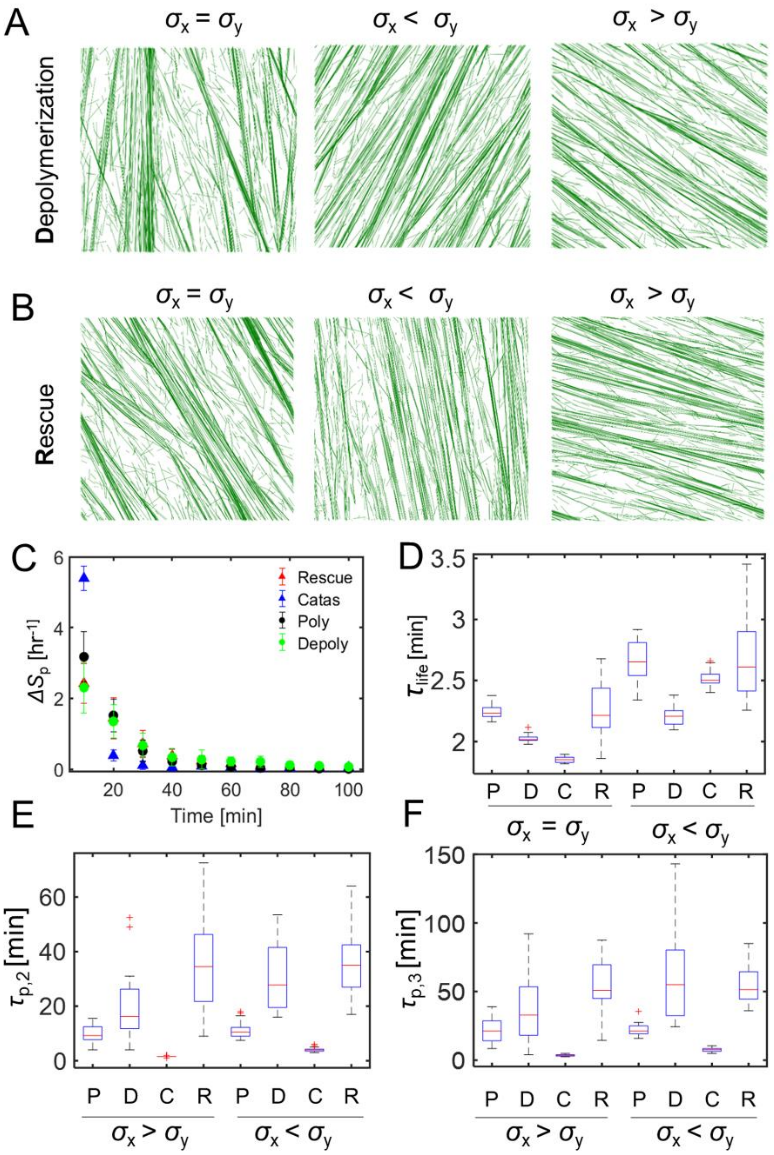

Figure S2. Anisotropic stress regulates network dynamics and microtubule lifetime. (A-B) Steady state MT network morphology. All snapshots are taken at 100 min. MTs are subject to network stress predominant in y direction (right), isotropic (left). In A and B, depolymerization rate is suppressed, and rescue frequency is enhanced in alignment with principal stress, respectively. (C) Time evolution of the rate of change in network order parameter for four different conditions with anisotropic stress. When there is a constitutive relationship between stress and catastrophe frequency, the efficiency of network alignment is significantly increased. In the case of polymerization rate, the increase is smaller. (D) Summary of the average lifetime of microtubule τ_{life} for all different conditions with isotropic vs. anisotropic stress in which principal stress influences individual stochastic parameter independently. (E-F) Boxplot of the time constants (second and third) acquired from cases with anisotropic stress predominant in x or y directions in all conditions. There is no significant difference due to directional of the principal stress. Data for each condition are averaged over 20 simulations. P: polymerization, D: depolymerization, C: catastrophe, R: rescue.

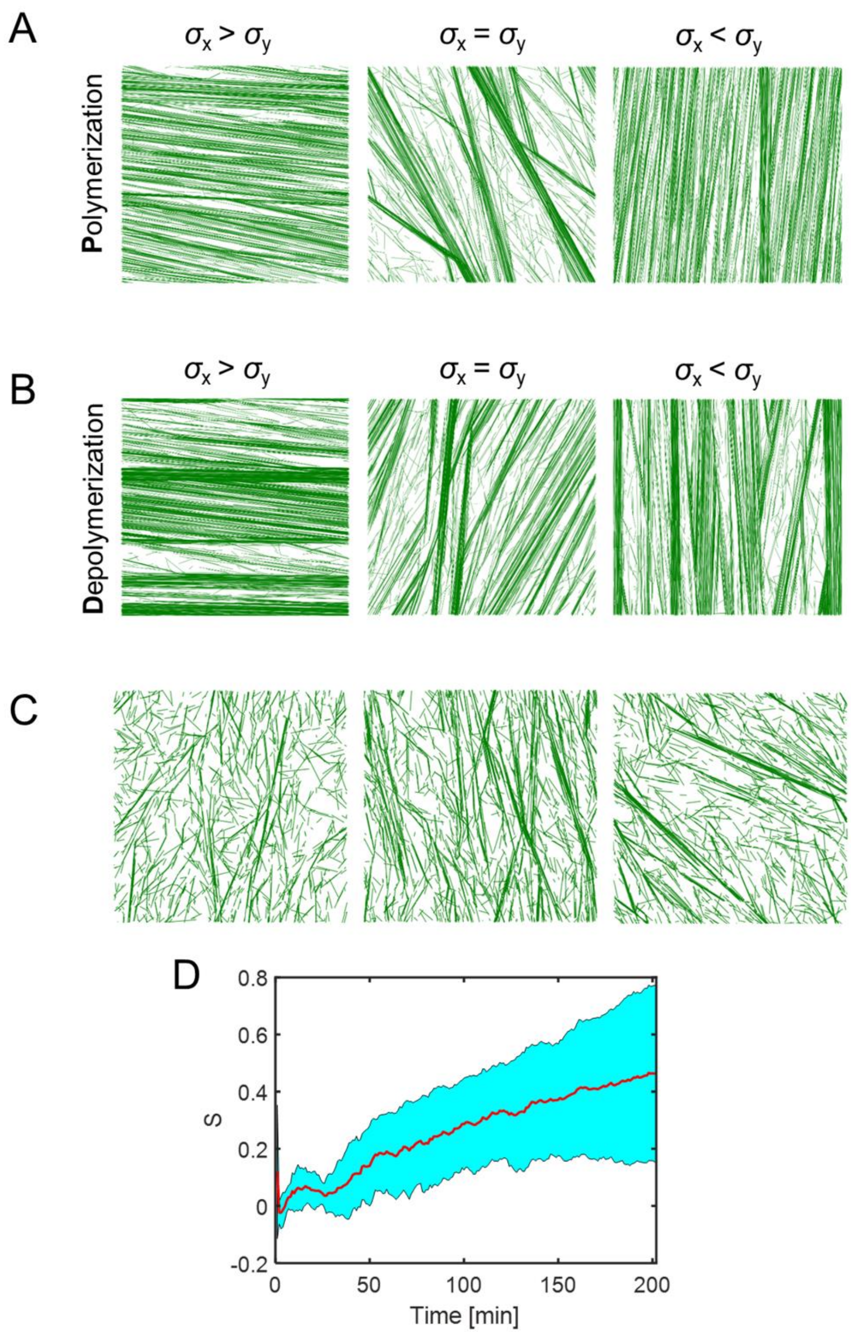

Figure S3. Simulations run with a larger computational domain or without stress. (A-B) Reference condition and stress-based simulation with larger network domain. Steady state MT morphology for network with size of 20 μm×20 μm. All snapshots were taken at 100 min. MTs are subjected to network stress predominant in y direction (right), isotropic (middle), predominant in x direction (left). In A and B, the polymerization rate is enhanced, and the depolymerization rate is suppressed in alignment with principal stress, respectively. (C-D) Representative cases showing steady state MT morphology for network with size of 10 μm×10 μm without stress. (C) All snapshots were taken at 100 min. (D) Time evolution of order parameter (N = 20 cases) with average (red) and standard deviation (cyan area). The order parameter is calculated with respect to a dominant angle.

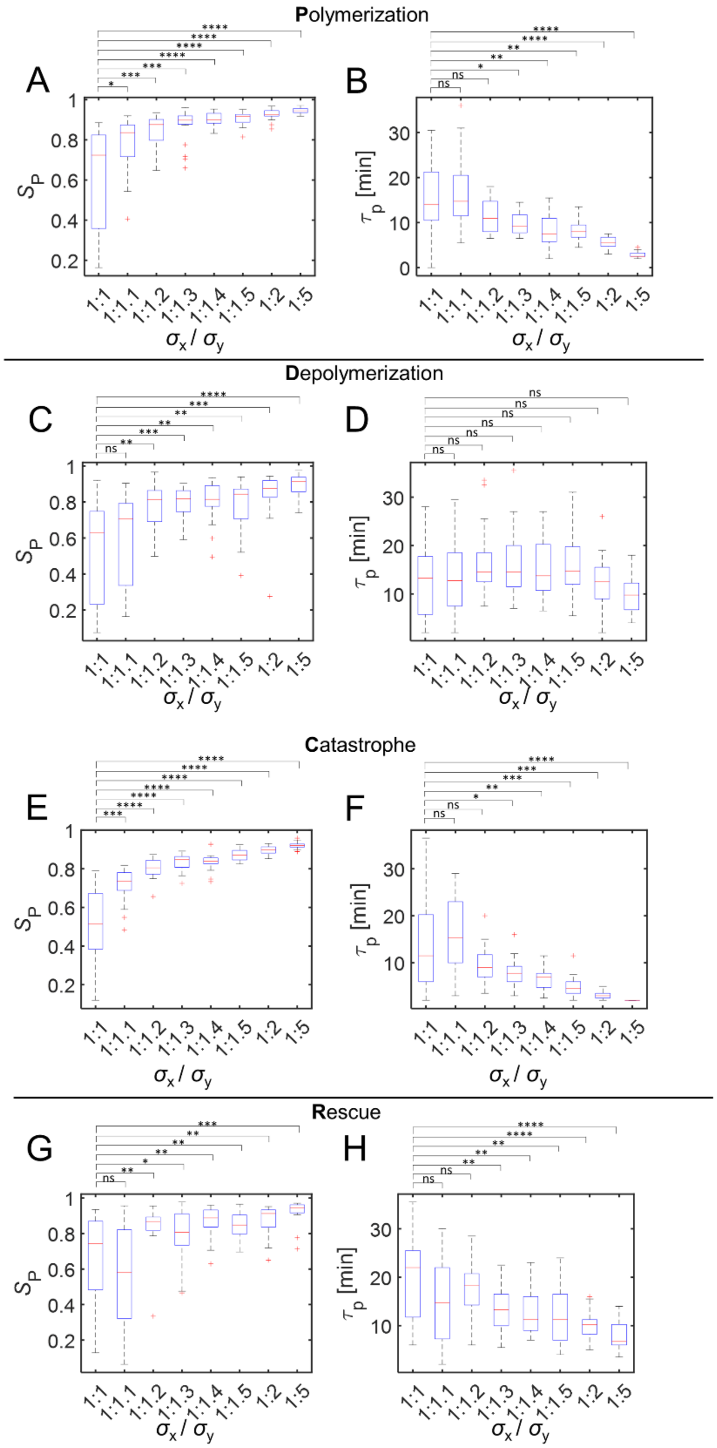

Figure S4. Effect on the microtubule ordering by ratio of anisotropic stress in the network. Isotropic stress has a ratio of 1:1. The anisotropic stress ratio is increased to 1.1, 1.2, 1.3, 1.4, 1.5, 2 and 5. (A, C, E, G) The order parameter of network at steady state as a function of stress anisotropy. (B, D, F, H) The first time constant of network order parameter as a function of stress anisotropy. Boxplot for each order parameter and time constant are acquired from 10 simulations in each case. P: polymerization (A,B), D: depolymerization (C,D), C: catastrophe (E,F), R: rescue (G,H); ns $p>0.05$, * $p<0.05$, ** $p<0.01$, *** $p<0.001$, **** $p<0.0001$.

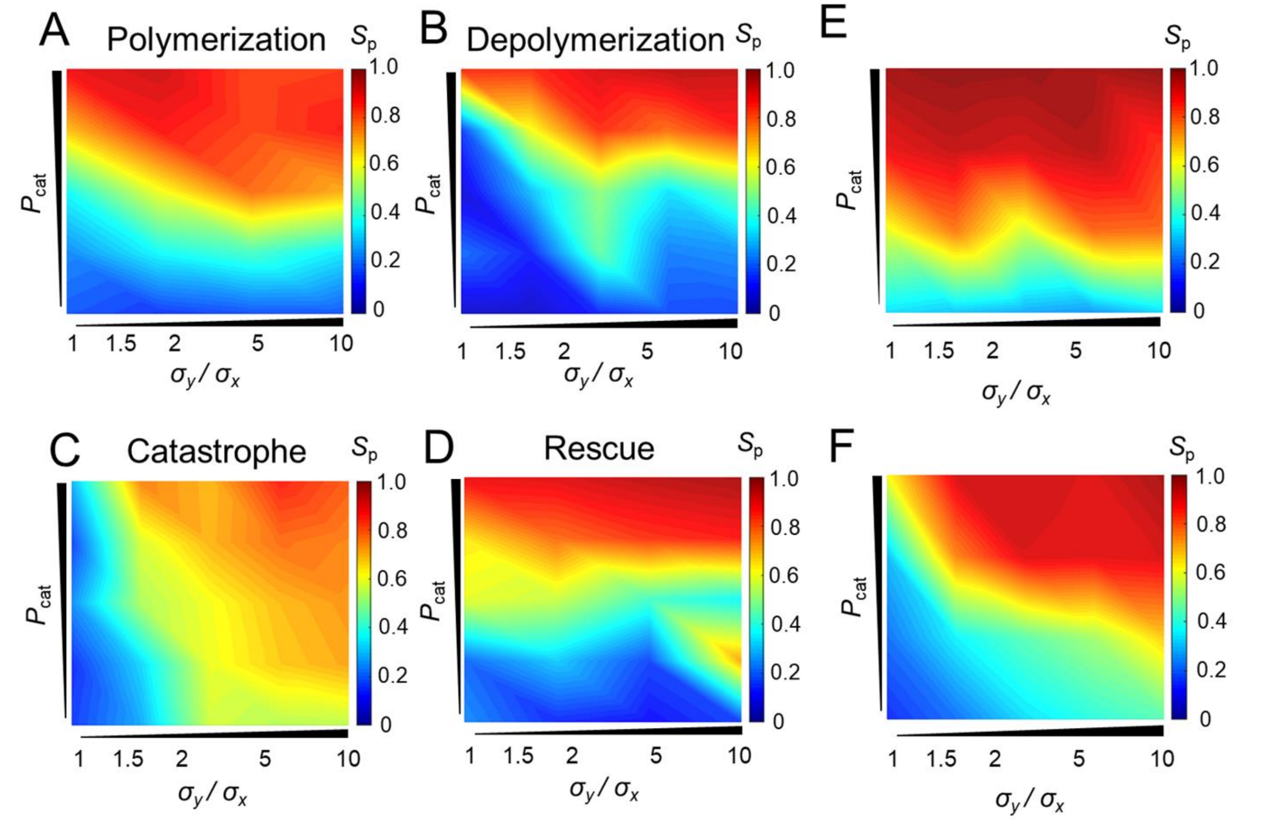

Figure S5. Stress anisotropy and collision-induced catastrophe coregulate microtubule ordering. In the cases when there is a constitutive relationship between stress and stochastic dynamics of microtubule, a two-dimensional parameterization is performed. (A-D) Heatmap summarizing the order parameter of network at steady state for cases of polymerization (A), depolymerization (B), catastrophe frequency (C) and rescue frequency (D) when both stress anisotropy (x-axis) and collision-induced catastrophe probability P_{cat} (y-axis) are varied. Stress anisotropy ratio is varied from 1 to 1.2, 1.5, 2, 5, 10 and collision-induced catastrophe is varied from 0.2 to 0.4, 0.5, 0.8. (E-F) Stress anisotropy, collision-induced catastrophe and free catastrophe frequency show a combinative effect on network alignment. (E) Heatmap of network order parameter when base frequency of free catastrophe is elevated. Due to high free catastrophe frequency, the effect by anisotropy of stress (x-axis) is minimized. By increasing the collision-induced catastrophe probability P_{cat} (y-axis), the order parameter increases and remains independent of stress anisotropy. (F) Heatmap of network order parameter when base frequency of free catastrophe is reduced. The dependence of order parameter on stress anisotropy and P_{cat} are equal. Higher stress anisotropy with higher P_{cat} leads to the highest network alignment.

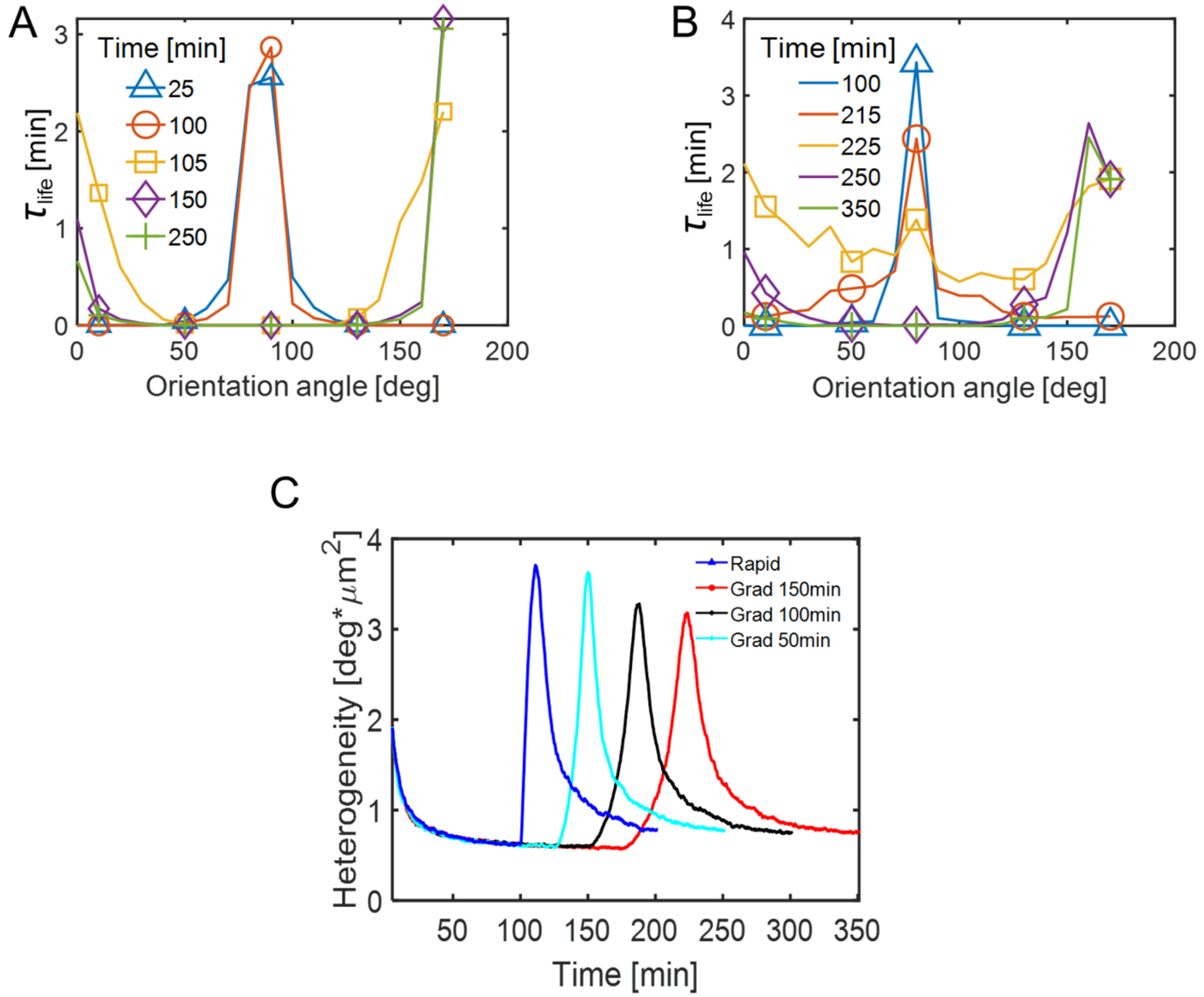

Figure S6. Microtubule dynamics during stress reorientation. (A, B) Distribution of average microtubule lifetime with different orientation angles when stress reorientation occurs rapidly (A) and gradually (B), corresponding to Figures 3A and B. In both cases, the initial orientation of microtubules is predominant near 90˚ (vertical) with much longer lifetime. After stress pattern reorientation ~100 min, the microtubules with longer lifetime shift toward 0˚ or 180˚ in the horizontal direction until the end of simulation. (C) Network heterogeneity index as a function of time for various stress pattern transition conditions. The heterogeneity is defined as the standard deviation of microtubule angles divided by the total density.

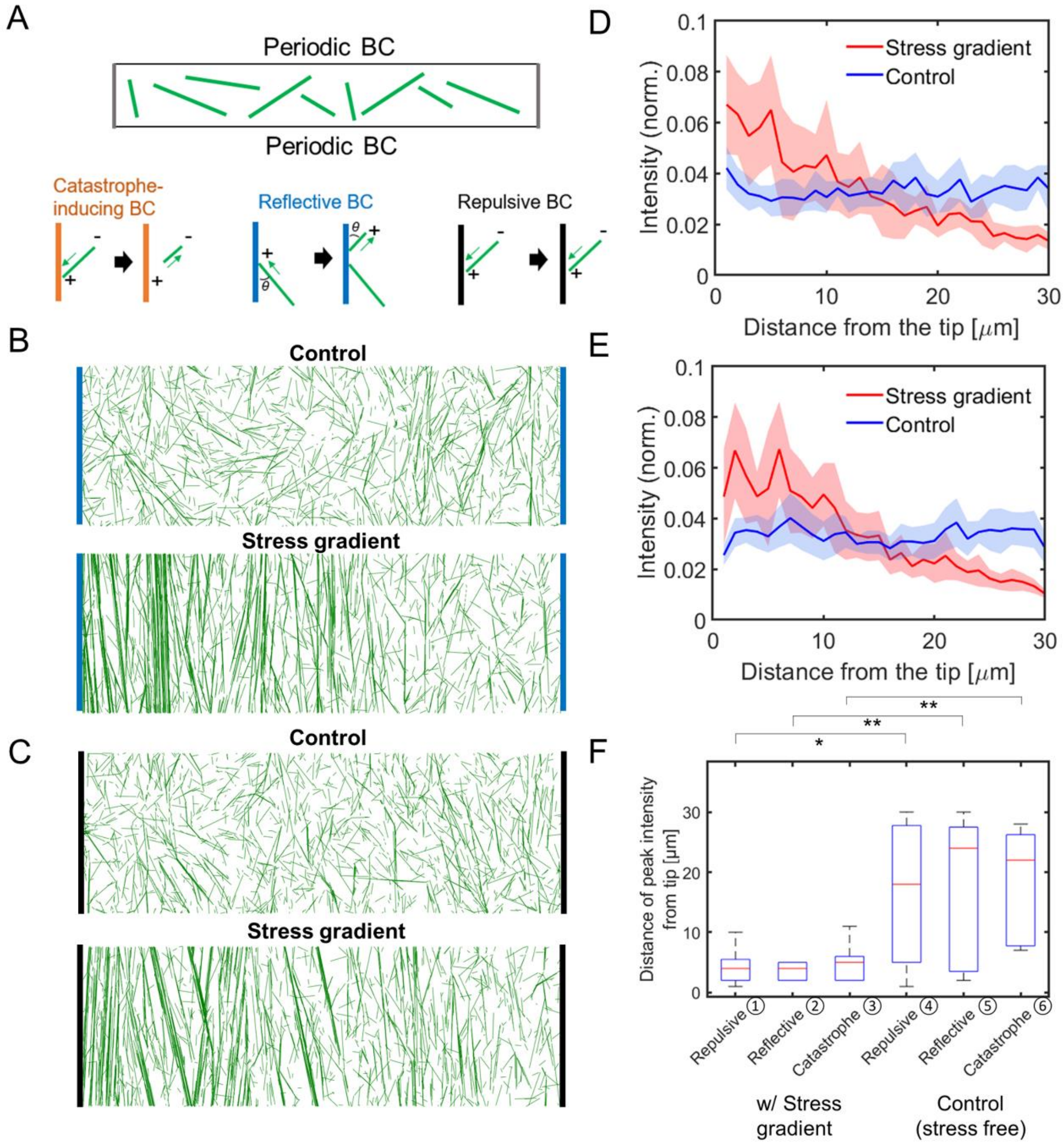

Figure S7. Network with various boundary conditions. (A) Schematic of the network. The long edges are assumed to have periodic boundary conditions. The left and right edges are subjected to three different types of boundary conditions. Catastrophe-inducing boundary is explained in Figure 4. Reflective boundary (blue): when a microtubule encounters a boundary, it keeps growing at the same angle as it hits the boundary in the new direction reflective to the old direction. Repulsive boundary (black): a microtubule hitting a boundary would switch to a pause state. (B, C) Network morphology at 100 min when no stress is included (upper panel) or with a stress gradient (lower panel) for reflective boundaries (B) or repulsive boundaries (C). (D, E) Steady state intensity distribution of microtubules in (B) and (C), with increasing distance from the tip. Without stress, the network is homogeneous. Distinct bundles near the cell tip can form transverse-band-like pattern with a stress gradient regardless of the boundary conditions. (F) Distance of the peak intensity of microtubules from the tip. With a stress gradient, density peaks near the cell tip as transverse bands form. Without stress, the increased homogeneity of network leads to random distribution of local peak intensity.

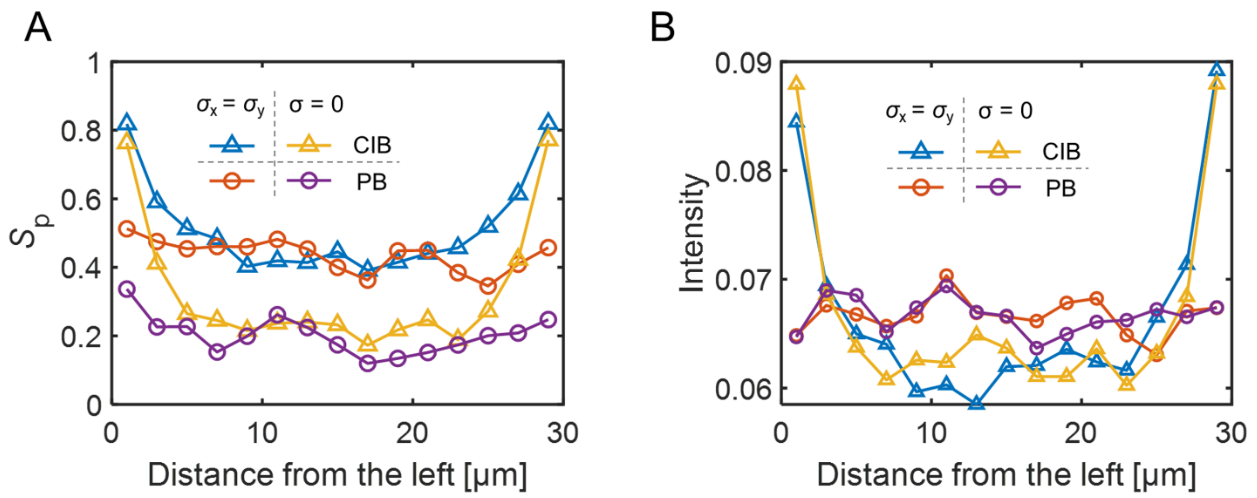

Figure S8. Isotropic stress combined with catastrophe-inducing boundary affects self-organization of microtubules and correlates with local orientation in larger domain. (A) Local order parameter as a function of the distance from the left boundary. (B) Local intensity of microtubules with increasing distance from the left boundary. Legend: Catastrophe-inducing boundary (CIB) with isotropic stress (blue triangle) or no stress (yellow triangle). Periodic boundary (PB) with isotropic stress (orange circle) and no stress (purple circle).

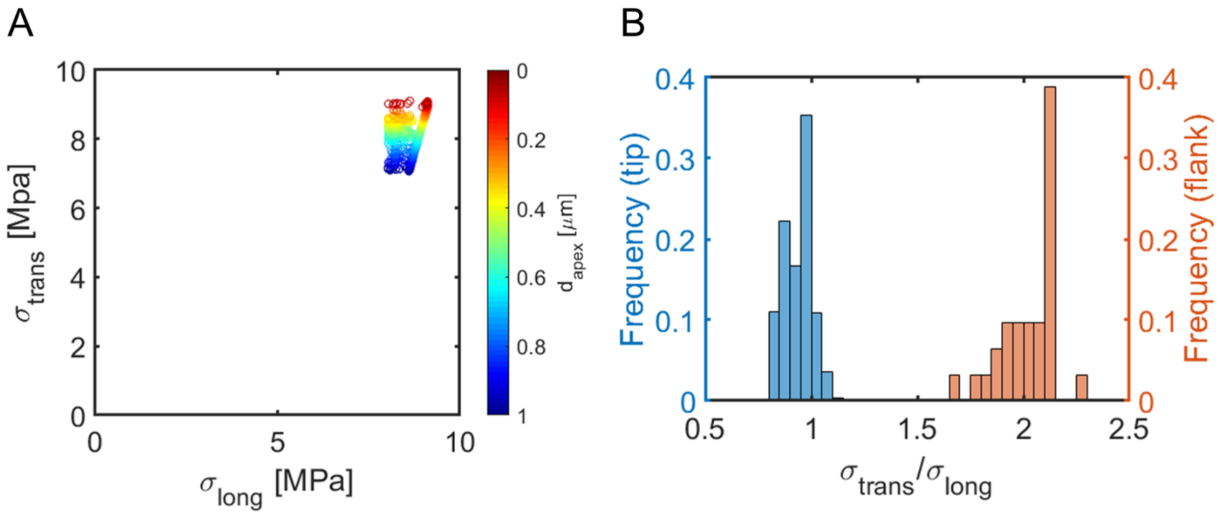

Figure S9. Stress profile from the FEM model. (A) Paired values of longitudinal and transverse stress in the apex of trichome model. Color indicates the distance from apex. (B) Distribution of the stress anisotropy ratio in the tip zone (blue) and flank region (orange). Stress is isotropic in the tip zone while anisotropic in the flank region, as implemented in our simulations.
